# A System for Genome-Wide Histone Variant Dynamics In ES Cells Reveals Dynamic MacroH2A2 Replacement at Promoters

**DOI:** 10.1371/journal.pgen.1004515

**Published:** 2014-08-07

**Authors:** Ozlem Yildirim, Jui-Hung Hung, Ryan J. Cedeno, Zhiping Weng, Christopher J. Lengner, Oliver J. Rando

**Affiliations:** 1 Department of Biochemistry and Molecular Pharmacology, University of Massachusetts Medical School, Worcester, Massachusetts, United States of America; 2 Program in Bioinformatics and Integrative Biology, University of Massachusetts Medical School, Worcester, Massachusetts, United States of America; 3 Department of Animal Biology, School of Veterinary Medicine and Institute for Regenerative Medicine, University of Pennsylvania, Philadelphia, Pennsylvania, United States of America; Ludwig Institute for Cancer Research, University of California San Diego, United States of America

## Abstract

Dynamic exchange of a subset of nucleosomes *in vivo* plays important roles in epigenetic inheritance of chromatin states, chromatin insulator function, chromosome folding, and the maintenance of the pluripotent state of embryonic stem cells. Here, we extend a pulse-chase strategy for carrying out genome-wide measurements of histone dynamics to several histone variants in murine embryonic stem cells and somatic tissues, recapitulating expected characteristics of the well characterized H3.3 histone variant. We extended this system to the less-studied MacroH2A2 variant, commonly described as a “repressive” histone variant whose accumulation in chromatin is thought to fix the epigenetic state of differentiated cells. Unexpectedly, we found that while large intergenic blocks of MacroH2A2 were stably associated with the genome, promoter-associated peaks of MacroH2A2 exhibited relatively rapid exchange dynamics in ES cells, particularly at highly-transcribed genes. Upon differentiation to embryonic fibroblasts, MacroH2A2 was gained primarily in additional long, stably associated blocks across gene-poor regions, while overall turnover at promoters was greatly dampened. Our results reveal unanticipated dynamic behavior of the MacroH2A2 variant in pluripotent cells, and provide a resource for future studies of tissue-specific histone dynamics *in vivo*.

## Author Summary

The ability of cells to remember the correct cell fate is at least partly dependent on how the genome is packaged. Embryonic stem (ES) cells, which have the ability to become any cell type in the body, are a particularly well-studied system for understanding how the packaging of the genome – chromatin – controls cell state. One of the more curious aspects of ES cell chromatin is its “hyperdynamic” nature, as the histone proteins that comprise chromatin have been reported to exchange rapidly on and off the DNA in these cells. Here, we report a pulse chase system for studying histone dynamics in mouse ES cells, and report on the dynamics of two histone variants, H3.3 and MacroH2A2. Notably, MacroH2A2 is highly dynamic in ES cells, with rapid exchange occurring over gene promoters, alongside much more stably-bound domains that cover large blocks of the genome. Upon differentiation to fibroblasts MacroH2A2 becomes much more stably-bound to the genome, consistent with the idea that this histone variant plays a role in “locking down” repressed regions the genome. These results provide further evidence for a key role of histone dynamics in control of cell state inheritance.

## Introduction

All genomic transactions in eukaryotes occur in the context of chromatin. While histones are generally among the most stably-associated DNA-binding proteins known [1], a subset of histones exhibit dynamic replication-independent exchange with the soluble pool of nucleoplasmic histones [2]–[4]. Dynamic histone exchange is intimately linked to a variety of key aspects of chromatin biology.

In all eukaryotes studied, histone H3 exchange is most rapid at promoters [5]–[12], and is generally slowest over heterochromatic regions. In addition, H3 exchange is rapid at boundary elements that block the spread of heterochromatin [5], [7], raising the possibility that rapid histone exchange could function mechanistically to erase laterally spreading chromatin states. These correlations, in which histone exchange is slow over epigenetically-heritable heterochromatin domains but is rapid at boundary elements, raise the question of how histone dynamics contribute to epigenetic inheritance. Interestingly, H3/H4 tetramers carrying the H3.3 variant “split” during replication to a greater extent than do H3.1-containing tetramers [13], consistent with the hypothesis that dynamic regions of chromatin could potentially self-perpetuate through replication [2]. In addition, the rapid histone turnover observed at promoter regions of actively transcribed genes suggests that histone turnover may have an important role in gene regulation, as higher histone turnover rates could provide greater access of regulatory proteins to specific DNA elements. Yet much remains to be learned about the mechanistic basis for, and the biological consequences of, dynamic chromatin states.

Embryonic stem (ES) cells are a key model for mammalian pluripotency and cell state inheritance. ES cells are characterized by unusual chromatin packaging [14], and a wide variety of chromatin regulators have been implicated in control of pluripotency and differentiation [15]–[19]. One curious feature of ES cell chromatin is its “hyperdynamic” state—photobleaching experiments show that many histone variants exchange more rapidly in ES cells than in differentiated cell types [20]. This hyperdynamic state has been proposed to maintain the ES cell genome accessible as a relatively permissive ground state that becomes “locked down” during the process of lineage commitment and subsequent differentiation. Understanding histone exchange dynamics in ES cells, and during differentiation, is therefore of great interest for understanding the roles for chromatin in cell state inheritance.

The histone variant MacroH2A plays a key role in cell state stabilization in mammals. Mammals encode three MacroH2A variants, MacroH2A1.1 and MacroH2A1.2, which are alternatively spliced isoforms of a single gene, and the distinct gene product MacroH2A2. All three MacroH2A variants are distinguished by the presence of the unusual “Macro” domain fused to their relatively well-conserved H2A cores. It has been suggested that MacroH2A plays a role in fixing the epigenetic state of differentiated cells (reviewed in [21]). Support for this notion comes from observations that MacroH2A deposition increases with cellular age and senescence [22], [23], and that epigenetic reprogramming via somatic cell nuclear transfer is accompanied by an active removal of MacroH2A1 from the donor chromatin upon transfer into the ooplasm [24]. More recent studies have indicated that MacroH2A depletion from somatic cells increases their propensity for undergoing epigenetic reprogramming [25]–[28] —in several of these studies, depletion of either MacroH2A1 or MacroH2A2 enhances reprogramming, with depletion of both having an additive effect. These studies suggest that removal of MacroH2A from the somatic genome may be prerequisite for acquisition of pluripotency during epigenetic reprogramming.

MacroH2A may further contribute to fixing the epigenetic state of differentiated female cells due to its accumulation on the inactive X chromosome (Xi) [29]. However, association of MacroH2A1 with the Xi appears to occur after the random inactivation of the X chromosome (XCI) [30], and in conditional *Xist* deletions gene silencing is maintained despite the loss of MacroH2A1 on the Xi [31]. Nonetheless, while MacroH2A1 appears to be dispensable for XCI, removal of this variant from the Xi could still potentially represent a barrier to epigenetic reprogramming of a differentiated, post-XCI somatic cell to the pre-XCI ground state of pluripotency.

Despite the general characterization of MacroH2A as being a “repressive” histone variant, there are numerous examples where Macro incorporation is associated with increased gene expression, particularly during early lineage specification after embryoid body formation from ES cells [32], and more recently in embryonic fibroblasts where MacroH2A1 is present at high levels in the active *Thy1* gene, but nearly completely absent when this gene is silent in pluripotent ES cells [27]. Determining the dynamics of MacroH2A turnover in both pluripotent ES cells and somatic cells is therefore of paramount interest for gaining an in-depth understanding of the epigenetic processes underlying cellular reprogramming.

Three methods are currently used to study histone dynamics [33]. First, the original discovery that the H3.3 variant marks sites of replication-independent histone exchange [3], [4] has enabled many labs to infer histone dynamics simply from steady-state H3.3 localization patterns [6], [7], [9]–[11]. Second, genetically encoded “pulse-chase” systems have been utilized in which an epitope-tagged histone molecule is induced, and mapping of the epitope tag at various times after induction provides a detailed kinetic view of histone exchange dynamics [5], [8], . Finally, a metabolic labeling strategy termed “CATCH-IT” enables kinetic analysis of overall chromatin dynamics [37].

Here, we extend the approach of inducible expression of epitope-tagged histone variants to study chromatin dynamics in murine embryonic stem cells. We generated ES lines carrying doxycycline (“Dox”)-inducible HA-tagged versions of several histone variants, including H3.3 and MacroH2A2. These cells allowed us to monitor the rate of incorporation of HA-tagged variants by ChIP-Seq at varying times following Dox induction. For the well-studied H3.3 variant, we validate our method by recapitulating known aspects of H3.3 localization and dynamics. We also characterized the dynamics of the understudied MacroH2A2 variant in detail in ES cells and in their embryonic fibroblast (MEF) derivatives. MacroH2A2 exhibited broad, likely replication-coupled, incorporation throughout large stretches of the ES cell genome, along with unexpectedly rapid turnover behavior at highly-expressed promoters. In contrast, MacroH2A2 in more differentiated MEFs was additionally associated with a subset of gene-poor genomic loci, and its exchange at promoters slowed considerably. These results reveal surprising aspects of MacroH2A2 localization and dynamics and suggest that the view of MacroH2A2 as simply an indicator and/or mediator of repressed chromatin states is not accurate. Moreover, these studies establish a model system for investigation of histone variant dynamics in tissue culture systems as well as in complex organ systems *in vivo*.

## Results

### A system for genome-wide analysis of histone variant dynamics

**Figure 1:**
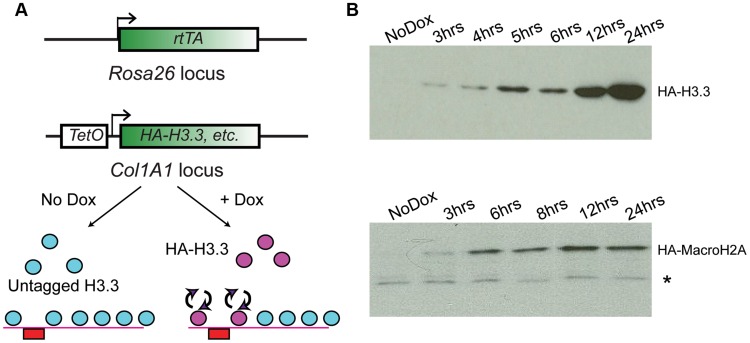
Generation of ES lines carrying inducible HA-tagged histones. (A) Schematic showing components of the tetracycline histone induction system. Below system components, arrows show cartoon schematic of the use of this system for assaying histone variant dynamics. (B) Western blot time courses for ES lines carrying Tet-inducible HA-H3.3 (top panel) or Tet-inducible HA-MacroH2A2 (bottom panel). * indicates crossreacting background band. See also **Figure S1**.

In order to assay genome-wide histone variant dynamics in embryonic stem cells and cell types derived from them, we generated ES cells based on the murine KH2 ES cell line [38], which harbors a modified reverse tetracycline transactivator (M2rtTA) targeted to the *ROSA26* locus and an FRT recombination site targeted into safe-haven chromatin downstream of the Type I Collagen (*Col1A1*) locus. Introduction of a donor plasmid carrying another FRT recombination site along with HA-tagged cDNA sequences encoding the histone variants of interest under transcriptional control of the tetracycline operator (TetO), along with an additional plasmid encoding the FLP recombinase, allows for site-specific integration of the tetracycline-inducible HA-tagged histone cassette into the genome (**Figure 1A**). Subsequent addition of the tetracycline analog doxycycline (“Dox”) to these ES cell clones or mice derived from them results in induction of the tagged histone variant (**Figure 1A**, bottom panel). Cell lines were generated and validated for several different histone variants, including MacroH2A2 (hereafter called Macro in some contexts), and H3.3 (**Figure S1A**).

For all cell lines analyzed, no expression of tagged histones was detected in the absence of Dox by Western blotting or immunofluorescence staining with an HA antibody. Robust activation of the tagged proteins was detectable within 2–3 hours of Dox addition (**Figure 1B** **, Figure S1B**). Several controls show that ectopic expression of tagged histone variants did not significantly perturb ES cell pluripotency. First, even after 12 hours of induction, ectopically expressed histones were far less abundant than the endogenous proteins levels (**Figure S1C**). Second, after 72 hours of overexpression, ES colonies maintained their pluripotent state as assessed by cell morphology, alkaline phosphatase (AP) staining, and expression of pluripotency markers such as Oct4, Sox2 and Nanog (**Figure S2**). The only exception was H3.3, where 72 (but not 24) hours of ectopic expression resulted in a slightly reduced proliferation rate, but did not compromise pluripotency based on Oct4 or AP staining (not shown). Finally, as shown below, mapping of total MacroH2A2 (for which a high quality commercial antibody exists) both before and after HA-Macro induction yielded nearly identical results, demonstrating that ectopic expression did not drive nonphysiological incorporation of this histone variant into ectopic sites throughout genome.

### Genome-wide dynamics of H3.3 replacement in mES cells

To validate our system, we first sought to determine whether a pulse-chase experiment is consistent with steady state mapping of H3.3 localization [4], [6], [7], [9], and what additional information it provides. ES cells carrying doxycycline-inducible HA-H3.3 were treated with Dox, and harvested after 0, 3, or 6 hours. HA-H3.3-containing chromatin was mapped genome-wide by chromatin immunoprecipitation followed by Illumina deep sequencing (ChIP-Seq). Sequencing reads were mapped back to the genome. Importantly, HA mapping at t = 0 (no doxycycline) did not show enrichment over specific loci but rather genome-wide nonspecific background, demonstrating the specificity of the anti-HA antibody (see below). Because H3.3 replacement is strongly associated with the 5′ ends of genes [6], [7], we aligned all annotated genes by their transcription start sites (TSSs), and averaged all mapped reads at each position relative to the TSS (**Figure S3**). Consistent with studies in flies and murine ES cells [6], [7], [9], we find that H3.3 is localized to two peaks surrounding the TSS, and that H3.3 levels correlate with the mRNA abundance of the associated gene. We also confirmed that the rapid H3.3 dynamics observed at Polycomb-bound regulatory elements in flies [7] are also present in the mouse embryonic stem cell genome at regions occupied by polycomb proteins Rnf2 and Suz12 (**Figure S3E**). Our results therefore recapitulate major known aspects of histone H3.3 dynamics. As H3.3 replacement has been extensively studied, we therefore turned to the understudied MacroH2A.2 variant.

### MacroH2A2 associates with gene-rich regions and promoters in ES cells

**Figure 2:**
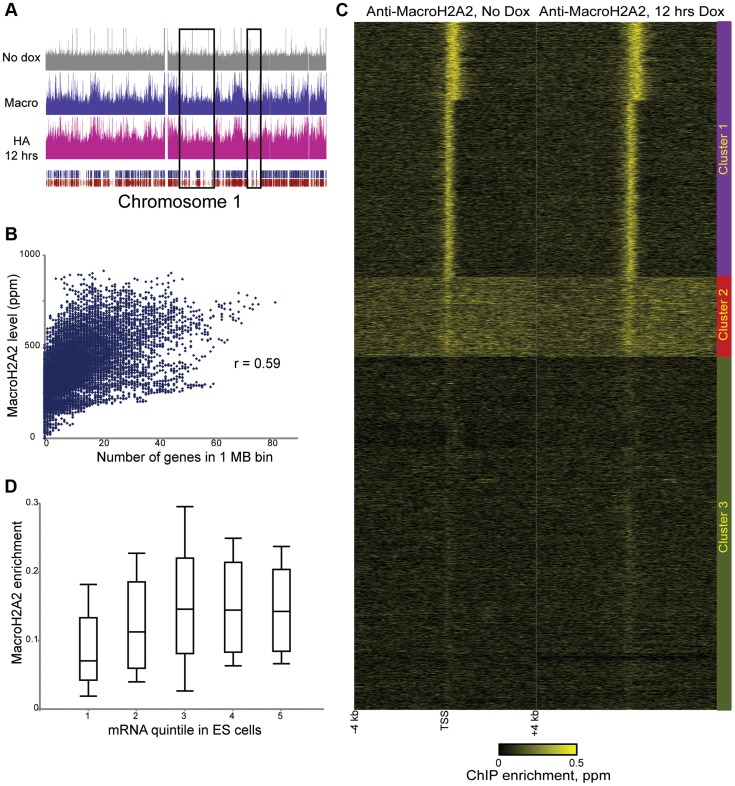
Steady-state mapping of MacroH2A2. (A) Overview of MacroH2A2 localization across chromosome 1. Shown are HA data from HA-Macro ES cells in the absence of doxycycline (top panel), or after 12 hours of doxycycline addition (in pink). Data from anti-MacroH2A2 is shown in blue. Rectangular blue and red tracks underneath the HA-12 hrs track represent RefSeq genes and Ensemble genes, respectively. Black boxes show gene “deserts” with relatively infrequent genes, which also exhibit reduced MacroH2A2 levels. (B) MacroH2A2 broadly localizes to gene-rich regions. The mouse genome was segmented into 1 Megabase tiles, and scatterplot shows strong correlation between gene number in a given tile (x axis) and overall MacroH2A2 signal in that tile (y axis). (C) TSS-aligned heatmaps for two related MacroH2A2 datasets. Note that left MacroH2A2 was generated from HA-Macro ES cells prior to doxycycline induction, while right panel represents t = 12 hours of doxycycline induction. All named genes are shown. Data was subject to k means clustering (k = 4), but given the strong similarities between two of the clusters we grouped them together into Cluster 1 leaving three clusters in this image. (D) Boxplot of MacroH2A2 enrichment for genes ranked by expression level [42]. Five categories on x axis show genes grouped from bottom 20% expression level in ES cells (left) to top 20% (right). For each quintile, MacroH2A2 enrichment (average enrichment level for 1.2 kb surrounding TSS) is shown as a boxplot, with box showing median and 1^st^ and 3^rd^ quartiles, and whiskers showing mean plus/minus standard deviation.

We next extended our studies to a histone variant with unknown dynamic properties, MacroH2A2. Because MacroH2A2 localization in ES cells has not been characterized, we first carried out genome wide mapping of MacroH2A2 in murine ES cells using a commercially available antibody (**Figure 2** **, Tables S1, S2**). MacroH2A2 was broadly localized to large (megabase-scale) blocks across the mouse genome, where it colocalized with regions of high gene density (**Figure 2A** **, Figure S4, Table S3**) —the correlation between average MacroH2A2 enrichment and gene density was 0.46 for 100 kb windows, and rose to 0.59 when considering 1 MB windows of the genome (**Figure 2B**). In addition to broad localization over gene-rich regions, we noted that MacroH2A2 exhibited a tight (∼500 bp) peak on average over promoters (**Figure 2C**). Counterintuitively, genes lacking MacroH2A2 were generally poorly expressed (**Figure 2D** and **Figure S4B**, see Cluster 3), stemming largely from the absence of MacroH2A2 at repressed gene families such as those encoding olfactory receptors or zinc finger transcription factors. Interestingly, among genes associated with promoter MacroH2A2, tighter localization was correlated with higher expression levels (**Figure S4B**, Clusters 1 and 2). Consistent with the surprising correlation between MacroH2A2 localization and active promoters, we found a moderately positive correlation between our MacroH2A2 dataset and H2A.Z localization [39] in ES cells (**Figure S5**).

Confidence in these surprising observations comes from four lines of evidence. First, localization datasets obtained before and after HA-MacroH2A2 induction were highly-correlated (**Figure 2C**). Second, anti-HA ChIP-Seq in uninduced HA-Macro cells yielded a nearly flat genome-wide background (**Figure 2A** **and Figure S4A**, top panel, **Figure S6**, left panel). Third, MacroH2A2 localization obtained using the MacroH2A2 antibody was very highly correlated with the localization pattern observed using anti-HA ChIP-Seq from cells expressing HA-MacroH2A2 (**Figure 2A** and **Figure S6**), but not HA-H3.3 (**Figure S3**). Finally, MacroH2A2 localization patterns were strongly correlated, but not identical, between ES cells and MEFs (see below). Thus, we find MacroH2A2 localizes to large blocks of gene-rich chromatin in ES cells, and within these blocks exhibits strong promoter localization at expressed genes.

### Rapid MacroH2A2 replacement at highly expressed promoters

**Figure 3:**
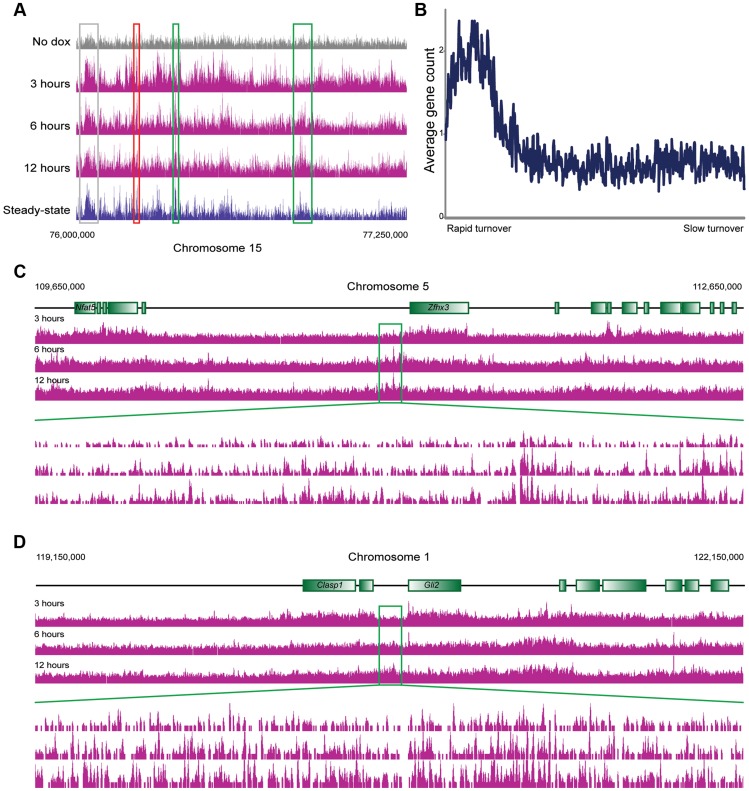
MacroH2A2 dynamics in ES cells. (A) Genome browser image for chromosome 15, 76,000,000–77,250,000. Anti-HA data are shown for no dox and for 3, 6, and 12 hours of HA-MacroH2A2 induction, as indicated, as well as mapping data from anti-MacroH2A2 ChIP. Boxes show regions of rapid (red), moderate (grey), or slow (green) MacroH2A2 replacement. Globally, correlation between anti-MacroH2A2 data and anti-HA data for t = 12 hours 100 kb bins (after removing unmappable bins and top 1% of artifactually-enriched bins) was r = 0.96. (B) Running window average of gene density (y axis) for 100 kb windows sorted according to inferred MacroH2A2 turnover (x axis) in ES cells. Inferred turnover is calculated as the log2 of t = 3 HA data divided by t = 12 HA data, and represents the Macro turnover rate *relative* to the genomic average. (C–D) Genome browser images showing long blocks of MacroH2A2 exhibiting relatively slow turnover dynamics. For each example, a 3 megabase genomic region is show in the top three panels, with a 100 kB zoom in shown in the 3 lower panels, as indicated. In these and many other cases, slow Macro turnover can be appreciated over 100 kb-scale domains that lack any annotated genes.

We next carried out genome wide mapping of HA-MacroH2A2 at 3 time points (3, 6, and 12 hours) after Dox induction. Reads were mapped back to the genome and genes were aligned by TSS as above. HA mapping in the no Dox control revealed a primarily flat genomic background (**Figures 2A** **,** **3A**), with trace levels of promoter localization likely resulting from low levels of leaky expression of HA-MacroH2A2 (**Figure S6**). Data from 3, 6, and 12 hours after HA-Macro induction was strongly correlated with endogenous MacroH2A2 localization (**Figure 3A** **, Figure S6**). The strong correlation between all 3 time points and the steady-state localization is to be expected from the fact that ES cells are rapidly cycling, so even at 3 hours of induction a substantial subpopulation of cells will have gone through S phase and carried out any replication-dependent MacroH2A2 incorporation.

In yeast, cell cycle arrest can be used to explicitly assay replication-independent histone replacement dynamics [5], [8]. However, this is impractical in ES cells, and our data come from asynchronously cycling cells. Nonetheless, such data can be used to study histone turnover. Two considerations, one conceptual and the other empirical, will aid in understanding how pulse-chase data obtained from cycling cells can be used to infer turnover dynamics (see **Figure S7**). Conceptually, we expect that loci exhibiting replication-coupled histone deposition (or slow replication-independent deposition) will gradually accumulate epitope-tagged histone variants over a time course of induction (**Figure S7A**). In contrast, because replication-independent replacement will initially occur in a greater fraction of cells than the subset of cells that are actively transiting S phase, such loci will exhibit more rapid accumulation of tagged histone. Given that genome-wide measurement methods typically normalize for sequencing depth (with the underlying assumption/hypothesis being equivalent total amounts of material between samples), the end result of this is that loci exhibiting rapid turnover will exhibit high levels of epitope tag enrichment early in a time course, but later in the time course this normalized *relative* enrichment will decrease as the bulk of cells transit S phase and replication-coupled deposition results in a greater total amount of epitope tag incorporated into the genome. In other words, relative enrichment of the rapidly exchanging population is high at early time points before population-wide assembly of HA-histone into the slower subpopulations, whereas at later time points normalization relative to the extensive HA-histone in cold domains results in a diminishing peak at “hot” loci (**Figure S7B**). Importantly, the assessment of relatively hot and cold loci is robust to normalization methods (**Figure S7B, Methods**). This predicted behavior is exactly what we have previously observed [5] *empirically* in yeast—here, replication-independent H3 turnover was directly measured in G1-arrested yeast. A parallel experiment was carried out using asynchronous cells, and those loci shown to exhibit rapid replication-independent turnover exhibited precisely the above-predicted behavior—rapid enrichment of tagged H3, followed by diminishing tag enrichment as the bulk of the genome was assembled into tagged H3 via replication-coupled assembly.

**Figure 4:**
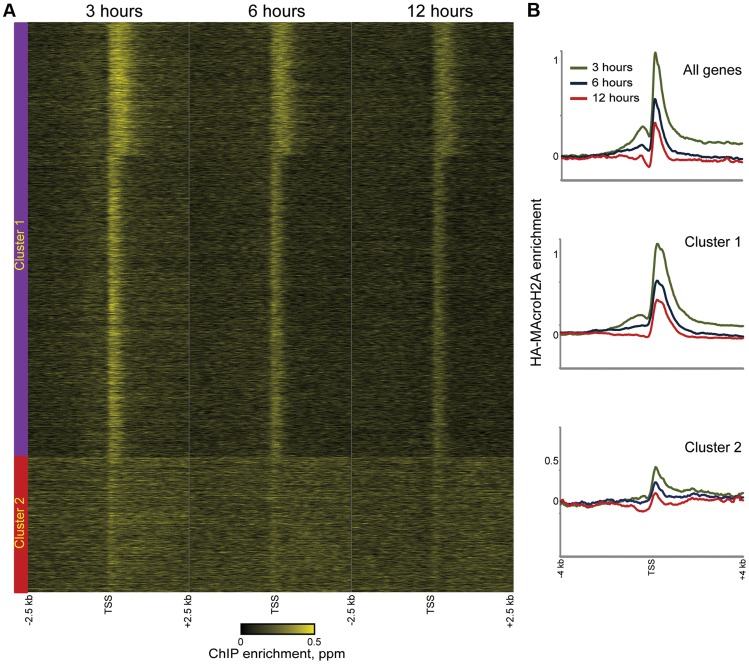
Rapid MacroH2A2 dynamics at promoters. (A) TSS-aligned heatmaps of genes from Clusters 1 and 2 (Clusters defined as in **Figure 2C**). In general, HA-Macro levels exhibit little change across the time course for Cluster 2, whereas Cluster 1 genes generally show diminishing *relative* HA enrichment over time. Heatmaps span 5 kb surrounding annotated TSSs. (B) Averaged data for all genes or genes in Cluster 1 or 2, for each of the 3 time points of HA-Macro induction. Data are renormalized for each group to the 1 kb spanning −2.5 kb to −1.5 kb, and y axis shows log(2) of the ratio between any given point and the upstream average.

Consistent with the above considerations, in addition to the genome-wide HA incorporation observed at all 3 time points, we also observe extensive locus-specific variation in HA-Macro dynamics (**Figure 3A**, red and green-bordered boxes identify regions of rapid and slow HA incorporation, respectively). Regions exhibiting high levels of HA at 3 hours relative to 12 hours were inferred to be “hot” (**Figure S7**, [5]), and typically occurred in highly delimited peaks associated with promoters (see below), whereas cold regions generally covered broad chromosomal stretches, often in intergenic regions (**Figures 3B–D** **, S8, Table S2**). These trends can also be seen in detail when focusing on promoter proximal Macro dynamics (**Figure 4**). On average, the TSS-proximal peak of MacroH2A2 diminished from 3 hours to 6 hours to 12 hours, consistent with rapid replication-independent replacement. This observation was reproduced in a second HA-Macro induction time course (**Figure S9**). In contrast, genes associated with broad domains of MacroH2A2 across their promoters (**Figures 2B** **and** **4A**, Cluster 2) exhibited consistent HA-MacroH2A2 mapping patterns at all three time points, as would be expected if these broad domains were relatively stable and incorporated Macro either via slow replacement or only during replication.

These results are consistent with at least two populations of MacroH2A2-containing chromatin that can be distinguished by their dynamic behavior. We infer that the TSS-proximal MacroH2A2 that is enriched at early time points before diminishing in enrichment represents a rapidly exchanging population of Macro that is present at moderate steady state occupancy, while larger Macro domains undergo either slow turnover or replication-coupled assembly. These larger domains tend to be gene poor, often occurring over gene deserts (**Figures 3C–D**) but occasionally encompassing individual genes as well (**Figure 4A**, cluster 2).

**Figure 5:**
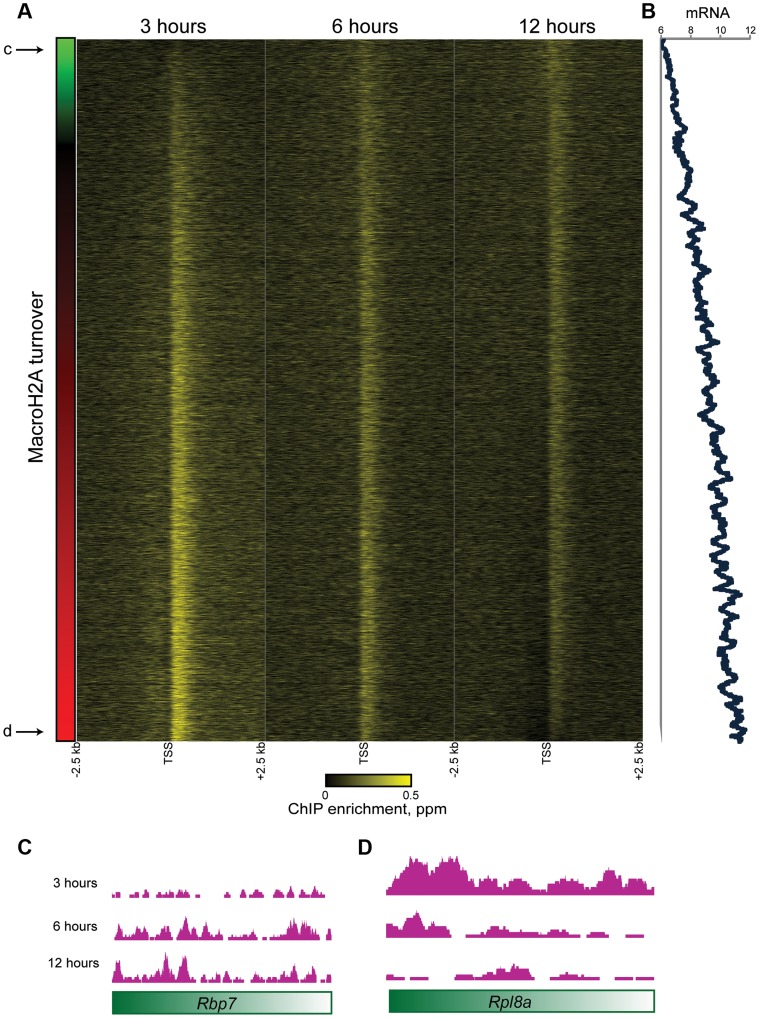
Promoter MacroH2A2 dynamics correlate with transcription. (A) Genes from Cluster 1 are sorted by the change in Macro enrichment from 3 to 12 hours (change in Macro is schematized in green to red colorbar to the left). (B) 80 gene running window average of mRNA abundance [42], expressed as log2 of the Affymetrix microarray probe intensity, for genes sorted as in (A). r = 0.46 between turnover (expressed as log2 t = 3/t = 12) and mRNA abundance. (C–D) Genome browser tracks for one “hot” and one “cold” example gene, as indicated in (A).

To gain further insight into the population of dynamic promoter-proximal Macro, we sorted genes with tight promoter Macro peaks (Cluster 1) according to their relative inferred Macro dynamics (**Figure 5A**) —note that *relative* dynamic behavior is completely insensitive to whether data are normalized assuming equivalent levels of Macro, or taking increasing total Macro incorporation over time into account. Genes with rapidly exchanging MacroH2A2 were enriched for GO processes consistent with housekeeping functions such as “translation” or “metabolism” (not shown) that are generally highly expressed, suggesting a potential link to expression level. Indeed, we found a strong correlation (r = 0.46) between MacroH2A2 dynamics and mRNA abundance (**Figure 5B**), as poorly expressed genes were associated with more stable MacroH2A2 than were highly expressed genes (see **Figures 5C–D** for examples). This link between promoter Macro dynamics and mRNA abundance supports our hypothesis that a pattern of diminishing HA enrichment over our time course is diagnostic of rapid MacroH2A2 replacement. These results are also consistent with the rapid histone H3 dynamics at promoters observed in a variety of organisms.

What is the function of rapid MacroH2A2 replacement at highly-expressed promoters? Knockdown of MacroH2A2 resulted in extremely modest changes in global mRNA abundance (**Table S4**), likely reflecting compensatory gene regulation by MacroH2A1, which is present in ES cells at ∼10-fold higher abundance than is MacroH2A2. Nonetheless, mRNA abundance exhibited greater changes at genes associated with slow MacroH2A2 exchange dynamics than at genes with rapid MacroH2A2 replacement (**Figure S10**).

### MacroH2A2 localization and dynamics in differentiated cells

Given that several histone variants are “hyperdynamic” in ES cells [20] and that the Macro content in somatic cells is considered an epigenetic barrier for epigenetic reprogramming to pluripotency [24], [26], we sought to characterize the changes in MacroH2A2 dynamics between ES cells and mouse embryonic fibroblasts (MEFs). We generated transgenic mouse embryos by injecting *TRE-HA-MacroH2A2* ES cells into blastocysts, derived MEFs from E12.5 chimeric embryos, then purified a homogenous population of *TRE-HA-MacroH2A2* MEFs after selection against host blastocyst-derived cells. Importantly, HA-Macro protein induction dynamics were similar in ES cells and MEFs (**Figure S11**), enabling comparisons of Macro dynamics using this system. We first mapped MacroH2A2 in MEFs using an anti-MacroH2A2 antibody. As observed for ES cells, Macro localization patterns were strongly correlated before and after Dox induction (**Figure S12**), and were strongly correlated with HA mapping data from Dox-induced cells (r = 0.98, see below), providing strong evidence for antibody specificity.

**Figure 6:**
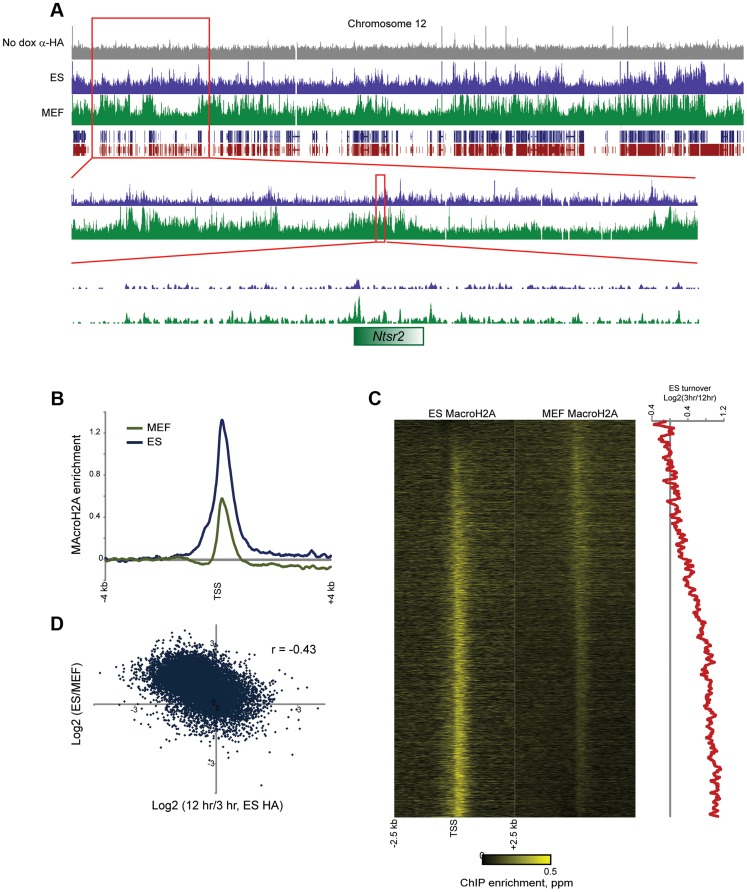
MacroH2A2 localization changes in embryonic fibroblasts. (A) Genome browser image for anti-HA control, MAcroH2A2 in ES cells, and MacroH2A2 in MEFs. Top panel shows chromosome 12, showing general agreement between Macro localization in ES cells and MEFs. Notably, some gene poor regions gain MacroH2A2 in MEFs relative to ES cells, as shown in two successive zooms. (B) TSS-aligned MacroH2A2 averages. All Anti-Macro mapping data are averaged for all named genes for ES cells and for MEFs, as indicated. Each trace represents two averaged replicate datasets. (C) Heatmaps showing MacroH2A2 promoter localization in ES cells and MEFs, as indicated. Note that data here are normalized relative to the average MacroH2A2 level in the corresponding cell type (rather than to genome-wide data). Genes are sorted by the difference in MacroH2A2 levels between ES cells and MEFs. Right panel shows 100 gene running window average of Macro turnover in ES cells (calculated as log2 of HA enrichment at t = 12 hrs vs/t = 3 hrs – high values represent slow turnover, low value represent rapid turnover). (D) Scatterplot of change in Macro enrichment between ES and MEFs (y axis) vs. Macro turnover in ES cells, calculated as in (C) (x axis).

Overall, MacroH2A2 patterns were similar (r = 0.67 using 100 kb bins) between ES cells and MEFs (**Figure 6A**, **Figure S13**, **Tables S1–S2**), supporting prior reports showing good correlations for MacroH2A1 localization between different cell types [40]. There was a general increase in MacroH2A2-enriched regions in MEFs relative to ES cells (**Figure S13A**), consistent with the fact that MacroH2A2 levels are higher in MEFs than in ES cells. Overall, while MacroH2A2 was generally maintained at gene-rich regions in MEFs as well as ES cells (**Figure 6A** **, S13**), we identified a large number of additional regions that gained Macro in MEFs relative to ES cells. Interestingly, MEF-specific Macro domains typically occurred in gene-poor chromosomal regions (**Figure S13**). In terms of gene categories associated with the sparse genes found in these gene-poor regions, MEFs gained MacroH2A2 at a broad set of genes involved in alternative differentiation programs including neural, leukocyte, muscle, and spermatogenesis programs (**Table S5**). This broadly supports the idea that the more plastic pluripotent chromatin state becomes progressively restricted during differentiation, with unused genes in each variety of differentiated cell type becoming “locked down” via MacroH2A2 incorporation.

In addition to broad gains of MacroH2A2 over gene-poor regions, we observed widespread changes in Macro enrichment over promoters between MEFs and ES cells. The average peak of MacroH2A2 over promoters exhibited an apparent decrease in MEFs (**Figure 6B**), although given that genome-wide there is more MacroH2A2 signal distant from promoters in MEFs relative to ES cells, this loss is overestimated as a result of dataset normalization. Accounting for this possibility, we nonetheless noted extensive redistribution of promoter-localized MacroH2A2 between ES cells and MEFs (**Figure 6C**). Curiously, MacroH2A2 changes between ES cells and MEFs correlated poorly (r = 0.02) with gene expression changes between these cell types, although we did note that exceptionally unpregulated genes characteristic of fibroblasts such as collagen and extracellular matrix factors (*Col1a1*, *Col5a1*, *Lox*, *Tgfb2*, *Fib1*, etc.) generally lost MacroH2A2 at their promoters in MEFs (**Tables S1–S2**). Instead of correlating with gene expression changes, we found that loss of Macro in MEFs tended to occur at promoters exhibiting dynamic Macro turnover in ES cells (**Figures 6C–D**). In contrast, *stably* Macro-associated promoters in ES cells preferentially retained Macro in MEFs. Together, these results suggest that dynamic assembly and disassembly of MacroH2A2 at highly expressed promoters is a specific feature of ES cells that is lost upon differentiation. In other words, while ribosomal protein genes (*Rpl8*, *Rpl32*, etc.) are highly expressed in both ES cells and MEFs, in ES cells their promoters are associated with rapidly-exchanging Macro, whereas these promoters are depleted of Macro in MEFs.

**Figure 7:**
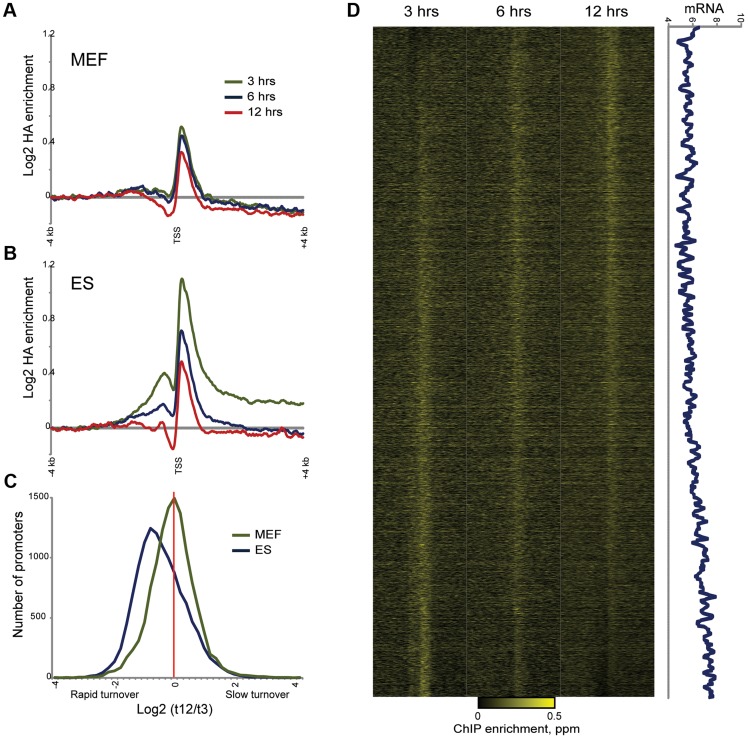
Altered MacroH2A2 dynamics in MEFs. (A) Averaged HA mapping data for MEFs during an HA-MacroH2A2 induction time course. Y axis is same scale as for (B), below. (B) As in (A), but for ES cells. Same as top panel in **Figure 4B**. (C) Distribution of promoter turnover behavior for MEFs and ES cells. For each cell type, we estimated turnover behavior using the log2 of promoter HA data for t = 3 divided by t = 12. For each cell type, histogram shows number of genes in each bin, from rapid turnover (negative values) to slow (positive values). Note the distinct left-shifted distribution for ES cells, characteristic of overall rapid promoter Macro dynamics. In addition to being centered on zero, the width of the distribution is tighter for MEFs, indicating less variation in promoter Macro turnover in these cells. (D) Heatmap view of HA mapping data from MEFs, sorted by inferred turnover from slow (top) to rapid (bottom). Right panel shows a 80 gene running window average of mRNA abundance (log2 of Affymetrix single channel intensity). r = 0.17 between MEF Macro turnover and mRNA abundance, as compared with r = 0.46 for ES cells (**Figure 5B**).

To explicitly characterize Macro dynamics in MEFs, we carried out HA-Macro mapping at 3, 6, and 12 hours after Dox induction. As with ES cells, HA localization at all 3 time points was highly correlated (r = 0.98 for all three time points using 100 kb windows) with mapping data obtained using the anti-Macro antibody. In contrast to ES cells, however, inspection of genome browser tracks yielded many fewer instances of 3 hour HA peaks that diminished at 6 and 12 hours. More systematically, we found that the average TSS-proximal HA peak was nearly identical at all three time points (compare **Figures 7A and B**). Not only was the average promoter HA peak nearly identical at all three time points, but there was less variation from t = 3 to t = 12 in our MEF data than in our ES data (**Figure 7C**). Sorting genes by inferred turnover behavior in MEFs revealed a subtle correlation between promoter turnover kinetics and mRNA abundance in MEFs (**Figure 7D**), but this relationship was far less robust (r = 0.17 versus r = 0.46) than that observed in ES cells (**Figure S14**). Taken together, these data show that rapid MacroH2A2 turnover is a specific feature of ES cells, and that upon differentiation to MEFs Macro is lost from dynamic promoters but retained in larger blocks of stably-associated Macro.

## Discussion

Here, we extended a genetically encoded pulse-chase approach to use inducible epitope-tagged histone variants to study chromatin dynamics in mammalian cells. The inclusion of a temporal component in studying histone variant turnover yields information normally lost when analyzing static binding data, even for the well-studied H3.3 histone variant. Our use of non-transformed pluripotent embryonic stem cells and primary mouse embryonic fibroblasts demonstrate the broad utility of this approach in mammals.

### MacroH2A2 localization and dynamics

We primarily focus here on the relatively unstudied MacroH2A2 variant. Overall, we observe extensive differences in the localization and dynamics of this variant between pluripotent ES cells and committed mouse embryonic fibroblasts. In ES cells, we observed widespread localization of MacroH2A2 across gene rich domains, along with a strong TSS-proximal peak of MacroH2A2. From our time course mapping studies, we infer that MacroH2A2 is rapidly replaced at promoters, and that this replacement is positively correlated with a gene's expression level. It is worth noting that H3.3 replacement is also rapid at promoters and correlates with mRNA abundance, indicating that in ES cells promoters exhibit rapid turnover of multiple histone variants.

Upon differentiation to embryonic fibroblasts, MacroH2A2 is broadly gained over gene poor domains, resulting in increased MacroH2A2 levels over genes associated with alternative differentiation programs such as neural or immune cell differentiation. Intriguingly, MacroH2A2 becomes far less dynamic in MEFs, and moreover MacroH2A2 is generally lost from those promoters where it is most dynamic in ES cells. Among other things, this observation argues that dynamic MacroH2A2 replacement inferred at highly-expressed genes in ES cells does not simply reflect nonspecific association of ectopically expressed histones with “open” promoters, as the highly expressed genes in MEFs exhibit far more subtle Macro dynamics than do the same genes in ES cells. Removal of the X chromosome from all key analyses (**Figure S15**) does not alter any of the conclusions regarding the change in Macro behavior between ES cells and MEFs, which is unsurprising as both cell types used in this study are male and thus data from the X chromosome reflects only the active X.

Together, these results are broadly consistent with the idea that pluripotent cells are characterized by “hyperdynamic” chromatin [20]. Interestingly, in contrast to the global hyperdynamic state observed by photobleaching for other histone variants, here we observe local, rather than global, dynamic MacroH2A2 behavior at a small fraction of loci—promoters of highly expressed genes. It will be interesting to identify factors contributing to ES-specific promoter MacroH2A2 dynamics in future studies.

### MacroH2A2 dynamics in cell fate determination

Our findings that the dynamics of Macro turnover decrease as pluripotent ES cells become developmentally committed, and that stable MacroH2A2 becomes incorporated in gene poor regions and at genes associated with alternative cell fates in MEFs, have implications for the interpretation of several recent studies suggesting that the MacroH2A content of somatic cells acts as a barrier to epigenetic reprogramming of the genome to a pluripotent state. It is widely appreciated that Macro content increases during cellular differentiation and ageing, and studies employing somatic cell nuclear transfer (SCNT, or cloning), revealed that somatic MacroH2A1 is actively removed from the genome prior to the acquisition of pluripotency [24]. These observations, coupled with the accumulation of MacroH2A on the Xi during the process of X chromosome inactivation in female cells, suggest that removal of MacroH2A from the somatic genome may facilitate, or even be a prerequisite for, reprogramming to pluripotency.

Indeed, a recent study found that depletion of MacroH2A1 and 2 from somatic cells prior to initiation of epigenetic reprogramming via the ectopic expression of Oct4, Sox2, Klf4, and c-Myc (the Yamanaka factors—[41]) greatly improved reprogramming efficiency [27]. This study implicated repression of pluripotency-associated genes (*Oct4*, *Sox2*) with high MacroH2A1 content in somatic cells as the epigenetic barrier, such that removal of MacroH2A from pluripotency-associated promoters might allow for the reprogramming factors to more readily activate these genes. While this may be a contributing factor, in general MacroH2A content does not strongly predict gene repression. For example, in MEFs MacroH2A1 is highly enriched at the active *Thy1* gene, but in ES and iPS cells, where *Thy1* is silent, MacroH2A1 is nearly completely absent [27]. Indeed, in ES cells we find that MacroH2A2 is associated with active promoters (**Figure 2**), further arguing against a simple model for a universally repressive function of MacroH2A. Instead, we speculate that stable association of MacroH2A (**Figure S10**), rather than average MacroH2A occupancy per se, is more likely to play a role in gene repression. Consistent with this idea, we observe Macro enrichment over *Sox2* in both ES cells and in MEFs, but in ES cells this gene is marked by rapid Macro replacement whereas Macro association is much more stable in MEFs (not shown).

Our findings that (1) dynamic incorporation of MacroH2A2 in gene-rich regions is correlated with highly active promoters, and that (2) stable MacroH2A2 incorporation in gene-poor regions (harboring genes associated with alternative cell fates) in MEFs is correlated with gene silencing, suggests that Macro removal during reprogramming may be most critical at these stable loci for re-establishing the “permissive” chromatin state characteristic of pluripotent cells.

### Extension to tissue-specific chromatin dynamics

To date, the majority of studies on histone dynamics have been carried out in cell culture systems. However, it will be of great interest to begin understanding the tissue-specific differences in chromatin dynamics *in vivo*, both under control conditions and in response to environmental perturbations. Thus, we generated a inducible histone variant mouse strain after blastocyst injection of the *TRE-HA-H3.3 ES* cell line and successful germline transmission of the R26-M2rtTA and *TRE-HA-H3.3* alleles (**Figure S16**). Administration of 2 mg/mL doxycycline in the drinking water of *TRE-HA-H3.3* mice resulted in HA-H3.3 induction in liver nuclear extracts (**Figure S16C**). These animals will therefore provide a unique and exciting resource for characterization of histone dynamics in different tissues and cell types, and provide a proof of principle for the application of our approach *in vivo*.

## Materials and Methods

### Ethics statement

All procedures involving mice were reviewed and approved by the Institutional Animal Care and Use Committee of the University of Pennsylvania (Animal Welfare Assurance Reference Number #A3079-01, approved protocol #803415 granted to Dr. Lengner) and were in accordance with the guidelines set forth in the Guide for the Care and Use of Laboratory Animals of the National Research Council of the National Institutes of Health.

### Targeting & stable cell line derivation

cDNA of various histone variants ((H2a-MMM1013-98478233; H2Az-MMM1013-9498090; MacroH2a2-MMM1013-9201250; H3.3-MMM1013-98478016, H1o-MMM1013-65296, Open Biosystems & human H3.1-Kind gift of Eric Campeau)) were initially subcloned in-frame with the HA-tag, then were cloned into the unique *EcoRI* restriction site of the pBS31 vector containing a *PGK* promoter followed by an ATG start codon and an FRT recombination site, followed by a splice acceptor-double polyA cassette, the tetracycline operator with a minimal CMV promoter, the unique *EcoRI* site, and an SV40 polyadenylation signal. The pBS31 vector containing the histone/histone variant cDNA was then electroporated along with a Flpe recombinase-expressing vector into KH2 embryonic stem cells harboring the modified reverse tetracycline transactivator (M2rtTA) targeted to and under transcriptional control of the *ROSA26* locus, as well as an FRT-flanked *PGK-neomycinR* cassette followed by a promoterless, ATG-less hygromycinR cassette targeted downstream of the *Collagen1a1* locus [38]. Selection for hygromycin resistance upon flip-in yielded numerous colonies which were verified for proper site-specific recombination at the *Coll1a1* locus by digestion of genomic DNA and Southern blotting with a 3′ internal probe, yielding a 6.2 kb wildtype band, a 6.7 kb band for the FRT-containing knock-in allele, and a 4.1 kb band for the successfully flipped-in inducible allele. Together, the components of this system enable tetracycline induction of the epitope-tagged histone variant of choice in embryonic stem cells from a genomically-integrated construct.

### HA-histone induction

Activation of the TetOn HA-tagged histone expression was carried out by addition of 2 µg/mL doxycycline hyclate (Sigma D9891) to the culture media. Cells were collected at different induction time points and induction of HA tagged histone variants in ES cells was assayed via Western blot.

### Validation of ES cell pluripotency

ES cell cultures were fixed in 4% paraformaldehyde for 5 minutes prior to staining for pluripotency markers alkaline phosphatase and Oct4. Alkaline phosphatase was detected by enzymatic reaction using a Vector Red substrate kit (Vector Labs). Immunofluorescence staining for Oct4 was carried out by first permeabilizing and blocking in 5% FBS, 0.1% Triton-X 100 for 15 minutes, then incubating with an anti-Oct4 primary antibody at 1∶100 for 1 hr at room temperature (Rabbit polyclonal H-134, Santa Cruz Biotech). After 3 washes with PBS, cells were incubated with an anti-rabbit secondary antibody labeled with Cy3, washed, stained with DAPI for total DNA, and imaged.

### Chimera generation and MEF Isolation

HA-MacroH2A2 or HA-H3.3-inducible ES cells were injected into BDF2 blastocysts and transplanted into pseudopregnant recipient females. For HA-MacroH2A2 MEF isolation, pregnant females were euthanized at E12.5, embryos were dissected followed by removal of internal organs. Embryos were then minced in the presence of 0.25% Trypsin-EDTA and incubated at 37°C for 20 minutes. MEF medium was then added and cell suspension was titrated followed by plating cells onto two 15 cm culture dishes per embryo. Cells were cultured for 12 hours at 37°C, 3% CO_2_, and 5% O_2_ after which puromycin was added to the MEF culture media to select against host blastocyst-derived cells (by virtue of a constitutively active puromycin resistance cassette targeted to the *ROSA26* locus along with the M2rtTA). After 48 hours of puromycin selection, homogenous populations of HA-MacroH2A2 MEFs were trypsinized and frozen at passage 1. ChIP-Seq experiments on MEFs were carried out after thawing and one additional passage (i.e., p2 MEFs). For generation of HA-H3.3 mice, blastocyst injection was performed as above, but embryos were carried to term. High contribution chimeras (>95% by coat color) were backcrossed to Bl/6 mice to establish an inducible HA-H3.3 mouse colony.

### Time course & ChIP-Seq

ES cells were grown in standard ES media containing Lif (ES Gro, Millipore) on mitotically inactivated feeder MEFs until approximately 80% confluence. ES cells were then pre-plated on gelatin and incubated for 45 min to deplete feeder MEFs by virtue of their faster adherence than ES cells (roughly 3 hours). ES cells were then split onto three gelatinized plates each of which was induced at different time points by the addition of final 2 µg/mL doxycycline hyclate (Sigma). A similar procedure was used for induction of MEFs at passage 2. All time points were crosslinked with formaldehyde to a final concentration of 1% for 10 minutes, and were quenched with 125 mM glycine. Crosslinked cells were resuspended in 270 µl SDS-Lysis Buffer (1% SDS, 10 mM EDTA and 50 mM Tris-Cl, pH 8.1) including protease inhibitor complex (Sigma) and PMSF (Sigma), and chromatin was sonicated in Bioruptor (UCD-200) to an average size of 150–400 base pairs. 70 µg of chromatin of each time point was immunoprecipitated either with HA antibody (Abcam) or MacroH2A2 antibody (Abcam). Eluted ChIP materials were PCI (Phenol-Chloroform-Isoamylalcohol) extracted, RNAse (Qiagen) and CIP (NEB) treated.

### Deep sequencing library construction

ChIP material was then gel-purified and DNA fragments were blunt-ended and phosphorylated with the End-it-Repair kit (EPICENTRE). Illumina genome sequencing adaptors were ligated using the Fast-Link ligation kit (EPICENTRE) after the addition of adenosine nucleotide, using exo- Klenow. And samples were PCR amplified with Illumina genomic DNA sequencing primers. PCR products (250 to 450 bp in size) were gel purified and sent for Illumina GA2 “Solexa” sequencing at the UMass Worcester deep sequencing core facility.

### Data availability

Data will be available at Gene Expression Omnibus, Accession #GSE57665.

### Mapping and normalization

Raw FastQ reads were first collapsed by their sequences while the occurrences were kept. We then mapped reads to the mm9 genome using bowtie allowing at most one mismatch in the alignment. Only one mapping was randomly picked by the -M 1 parameter setting for dealing with multimappers. Each aligned coordinate was extended toward its 3′ end to reach 150 bp length (although extension was clipped if it exceeded the length of the chromosome). We calculated the relative distance to the nearest TSS for all named genes, and for each TSS tallied the sum of read occurrences from 4 kb upstream to 4 kb downstream. The occurrences were normalized to p.p.m. and binned in 20 bp intervals. For TSS-centered averages (as in Figure 3C, for example) data were additionally normalized relative to the average of the first 2 kb (from −4 kb to −2 kb).

Importantly, for turnover analyses, relatively hot and cold regions are insensitive to the normalization method used—if we normalized all datasets to the hottest regions of the genome, rather than observing decreasing HA enrichment at promoters over time, we would observe very slow incorporation across the rest of the genome with increasing enrichment over time. However, in the absence of a true benchmark with known absolute occupancy (eg a set of promoters with 100% occupancy of MacroH2A2 at t = 3 hours), we choose to utilize standard genome-wide normalization and interpret our dataset with these considerations in mind.

### 100 kb tile-based data analysis

The mouse genome was segmented into nonoverlapping 100 kb tiles (eg chromosome 1 1–100,000, chromosome 1 100,001–200,000, etc.). For each tile, total normalized Macro or HA levels were calculated, and number of annotated TSSs was counted (using only TSSs for named genes). Tiles with the top 1% of signal in the anti-HA dataset from uninduced cells were discarded, as these typically covered regions adjacent to extensive repeats that show artifactual “enrichment” in all public datasets examined, including pre-ChIP input sequencing. For computing correlations between datasets, unmappable tiles with zero mapped reads were also removed.

### Promoter-based data analysis

For all named genes, data were aggregated into 20 bp bins from −4 kb to +4 kb surrounding the annotated TSS. These data were used for clustering and visualization throughout. In addition, we calculated a summary statistic based on total enrichment values for the 1.2 kb stretch from −600 to +600 bp—this value was used for analyses such as **Figures 5A**, **6D**, or **7C** (and related Supporting Figures). For comparisons between ES cells and MEFs, we used all genes with an average promoter MacroH2A2 enrichment of at least 0.1, in one of the two datasets, for the 1.2 kB surrounding the TSS.

## Supporting Information

Figure S1 Validation of ES lines carrying inducible HA-tagged histones. (A) Southern blots showing correct integration of HA-histone constructs at the *Col1A1* locus of KH2 cells. (B) Time course Western blots of HA-histone variant expression. As in **Figure 1B**. (C) Low levels of ectopic HA-H3.3 expression. Left and right sides show time courses of HA-H3.3 induction. Top panels show β-actin Western blots for loading controls, bottom panels show anti-HA (left) or anti-H3 (right) blots. Similar experiments were not feasible for Macro-H2A as the HA tag did not introduce a detectable mobility shift on western blot.
(TIF)

Figure S2 Ectopic HA-tagged histones do not affect ES pluripotency markers. (A) RNA levels of pluripotency markers. Q-RT-PCR for *Oct4*, *Sox2*, and *Nanog* before and after 20 hours of dox induction for the indicated histone variants. (B) Alkaline phosphatase staining, for uninduced and 72 hour induction for HA-H2A and HA-MacroH2A2.
(TIF)

Figure S3 H3.3 dynamics. (A–B) HA-H3.3 mapping data for 3 and 6 hours after HA-H3.3 induction. ES cells carrying tet-inducible HA-H3.3 were subject to 3 or 6 hours of doxycycline, as indicated. TSS-aligned data are shown for all named genes (A), sorted according to expression level in ES cells (B). (C–D) Dynamic aspects of histone H3.3 replacement. Here, TSS-aligned ChIP-Seq data for HA-H3.3 are averaged for genes in each of four expression categories. Notably, highly-expressed genes show symmetric H3.3 peaks at 6 hours but show stronger downstream peaks at 3 hours, showing that steady-state mapping of H3.3 obscures subtleties of chromatin dynamics. In this regard our data subtly disagree with CATCH-IT metabolic labeling studies, which show more rapid overall protein dynamics upstream of the TSS than downstream [43]. This discrepancy could arise from the fact that CATCH-IT identifies replacement dynamics for ALL DNA-bound proteins, and this dataset explicitly focuses on H3.3, or may result from the fact that Yang et al do not analyze formaldehyde-crosslinked chromatin, whereas we use formaldehyde crosslinking. In any case, our observation of more rapid H3.3 replacement downstream of the TSS is consistent with the greater number of short transcripts generated downstream of promoters relative to upstream in mammals [44]. These results imply that under steady state mapping conditions (e.g. Goldberg et al), or after extended induction in a pulse-chase system (eg at 6 hours), nucleosomes exhibiting moderate to high turnover rates become saturated with H3.3. (E) Averaged anti-H3.3 data for the indicated Dox induction times, averaged for 8 kb surrounding Suz12 binding peaks [45].
(TIF)

Figure S4 ES cell MacroH2A2 localizes to gene-rich regions. (A) As in **Figure 2A**, but for chromosome 8. (B) Histogram of mRNA abundances [42] for genes in each of the three clusters from **Figure 2C**.
(TIF)

Figure S5 Comparison of MacroH2A2 and H2A.Z localization in ES cells. (A) Data for all named genes is shown for MacroH2A2 (this study) and H2A.Z [39], with genes sorted by MacroH2A2 level. (B) Scatterplot of promoter H2A variant enrichments. Enrichment for each variant was calculated as the average ChIP-Seq enrichment across 4 kB surrounding the TSS.
(TIF)

Figure S6 MAcroH2A2 localization in ES cells. Six panels show MacroH2A2 localization, or control, sorted according to K means clustering of anti-MacroH2A2 ChIP-Seq (**Figure 2C**) in ES cells. Panels show anti-HA or anti-MacroH2A2 datasets, as indicated, in tet-HA-MacroH2A2 cells induced with doxycycline for varying times as indicated. Note strong correlations between data from anti-Macro mapping and anti-HA mapping in induced cells. Signal is generally far lower in uninduced cells, although low level leaky expression presumably results in HA patterns similar to endogenous Macro localization. Alternatively, open chromatin may be more susceptible to artifactual isolation even in the absence of leaky HA expression.
(TIF)

Figure S7 Expected time course behavior in asynchronous cells. (A) Cartoon of a genomic locus in a population of cells during a time course of epitope-tagged histone expression. Untagged nucleosomes are colored blue, epitope tagged-nucleosomes are colored orange. Each time point shows four loci, meant to correspond to four different cells in a population. Over time, the locus undergoing replication-coupled histone variant incorporation gains epitope tag gradually as cells asynchronously transit S phase. In contrast, the locus exhibiting rapid turnover gains epitope-tagged histones even at early time points. (B) Predicted behavior of ChIP-Seq at the locus shown in (A). Thanks to genome-wide normalization methods, the “hot” locus will exhibit very high relative epitope tag enrichment at earlier time points, but this peak will diminish in amplitude as slow turnover or replication-dependent incorporation occurs in an increasing fraction of cells, yielding a greater total number of loci carrying the epitope tag. Importantly, assessment of relatively hot and cold loci is totally insensitive to normalization method—an alternative normalization could be used in which hot loci are assumed to be saturated at early time points, and in this case the right peak would not change and the left peak would show more dramatic increases in enrichment over time. Yet calculating turnover by comparing data from t = 3 and t = 12 would nonetheless show the exact same difference when comparing the kinetic behavior of the right peak with the behavior of the left peak.
(TIF)

Figure S8 Rapid MacroH2A2 turnover in gene-rich regions. Cumulative distribution of gene richness (genes/100 kb tile, y axis), for 100 kb tiles grouped by hottest (top 10%), coldest (bottom 10%), and intermediate (remaining) Macro turnover behavior.
(TIF)

Figure S9 Reproducible MacroH2A2 dynamics. For both panels, genome-wide anti-HA datasets are averaged for all named genes at 3, 6 and 12 hours post-HA-MAcroH2A2 induction. Left panel shows more recent dataset used for analyses throughout manuscript, right panel shows prior dataset (which was undersequenced, and hence not used for additional analyses). In both cases, progressive “loss” of Macro over TSSs reveals rapid Macro dynamics at promoters in ES cells.
(TIF)

Figure S10 MacroH2A2 represses genes with stable MacroH2A2. mRNA abundance changes resulting from MacroH2A2 knockdown were measured by Affymetrix microarray (**Table S4**). Change in mRNA abundance is plotted on the y axis (positive values represent mRNA derepression upon knockdown), and genes are sorted on the x axis by inferred MacroH2A2 turnover, from slow to rapid replacement. mRNA abundance changes are shown as an 80 gene running window average.
(TIF)

Figure S11 HA induction kinetics in ES and MEFs. Western blots showing anti-HA staining for the indicated time points of Dox induction in ES and MEF lines, as indicated.
(TIF)

Figure S12 Reproducible MacroH2A2 localization in MEFs. Genome-wide anti-Macro localization patterns for all genes (k means clustered, k = 4) for tet-inducible HA-Macro MEFS without Dox (left panel), or after 12 hours of Dox (right panel).
(TIF)

Figure S13 MacroH2A2 gain over gene-poor regions in MEFs. (A) Global changes in MacroH2A2 between ES cells and MEFs. Histogram of normalized MacroH2A2 enrichment (x axis) for 100 kb bins, shown for anti-MacroH2A2 ChIP-Seq from ES cells and MEFs, or for anti-HA ChIP-Seq from uninduced ES cells (grey). Note that anti-HA dataset is plotted on a different y axis scale (right). Increased right shift for MEFs is consistent with the known global gain in MacroH2A2 in this cell type relative to ES cells. (B) MacroH2A2 gain in MEFs primarily occurs in gene deserts. X axis shows changes in average MacroH2A2 enrichment between ES cells and MEFs – positive values represent gain in Macro in MEFs, negative values represent relative “loss” of Macro in MEFs. Y axis shows gene count per 100 kb window. Note that, due to increasing MacroH2A2 levels during differentiation (*H2afy2* is upregulated 4-fold at the mRNA level between ES cells and MEFs), it is likely that the apparent “loss” of Macro over gene-rich regions reflects maintenance of Macro levels, whereas the gain in Macro over gene deserts shown here is being underestimated. (C–E) Examples of gene-poor regions with greater levels of Macro in MEFs than in ES cells.
(TIF)

Figure S14 Macro dynamics are poorly-correlated with mRNA abundance in MEFs. (A–B) Scatterplot between inferred Macro dynamics (calculated as log2 of HA enrichment at 3 hours of Dox induction divided by HA levels at 12 hours), shown on the x axis, and mRNA abundance, on the y axis. Macro turnover in ES cells (A) shows a strong correlation between rapid turnover and high mRNA abundance, whereas this correlation is very weak in MEFs (B).
(TIF)

Figure S15 Macro differences between ES and MEFs do not result from X inactivation. (A–C) These panels reproduce **Figures 6C** **, S14B, and S14A**, respectively, but with all X-linked genes removed from the dataset. Other analyses are similarly unaffected by removal of X-linked genes.
(TIF)

Figure S16 Generation of turnover mice. (A) Left side shows schematic of ES injection into blastocysts. Right panel shows offspring generated from Tet-HA-H3.3 injection with coat color indicating very high level of chimaerism. (B) Southern blots of offspring showing germline transmission of both loci required for Doxycycline induction of HA-tagged H3.3. (C) Western blots of nuclear extracts prepared from livers of animals provided with Dox for the indicated times, showing expected induction of HA-H3.3.
(TIF)

Table S1 MacroH2A2 localization in ES cells. For each named TSS in the mouse genome, data show normalized MacroH2A2 enrichment (for two averaged replicate datasets) in ES cells for 20 bp bins from −1000 to +1000 relative to the TSS.
(XLSX)

Table S2 MacroH2A2 localization in MEFs. For each named TSS in the mouse genome, data show normalized MacroH2A2 enrichment (for two averaged replicate datasets) in MEFs for 20 bp bins from −1000 to +1000 relative to the TSS.
(XLSX)

Table S3 Tiling analysis for MacroH2A2 dynamics. The mouse genome was broken into overlapping 100 kb tiles, and normalized data are shown for each tile for MacroH2A2 localization (M2 antibody), or for HA enrichments at indicated times post-doxycycline induction for ES cells and MEFs. Note that for analyses in the paper, alternating tiles were discarded (eg only tiles 1–100,000, 100,001–200,000, etc.), and the tiles showing top 1% enrichment in the HA No dox dataset were also discarded.
(XLSX)

Table S4 Gene expression changes in MAcroH2A2 knockdown ES cells. Affymetrix mRNA abundance data for replicate experiments of control or anti-MacroH2A2 knockdown ES cells.
(XLSX)

Table S5 GO terms enriched among genes gaining MacroH2A2 in MEFs. Funcassociate-reported GO terms enrichment for genes showing greater MacroH2A2 enrichment in MEFs relative to ES cells (using a 1.2 kb value encompassing the TSS).
(XLSX)
