# Supplementary figures and images for: A System for Genome-Wide Histone Variant Dynamics In ES Cells Reveals Dynamic MacroH2A2 Replacement at Promoters
Source: PLoS Genet. 2014 Aug 7;10(8):e1004515. doi: 10.1371/journal.pgen.1004515 (PMC4125097; doi:10.1371/journal.pgen.1004515)

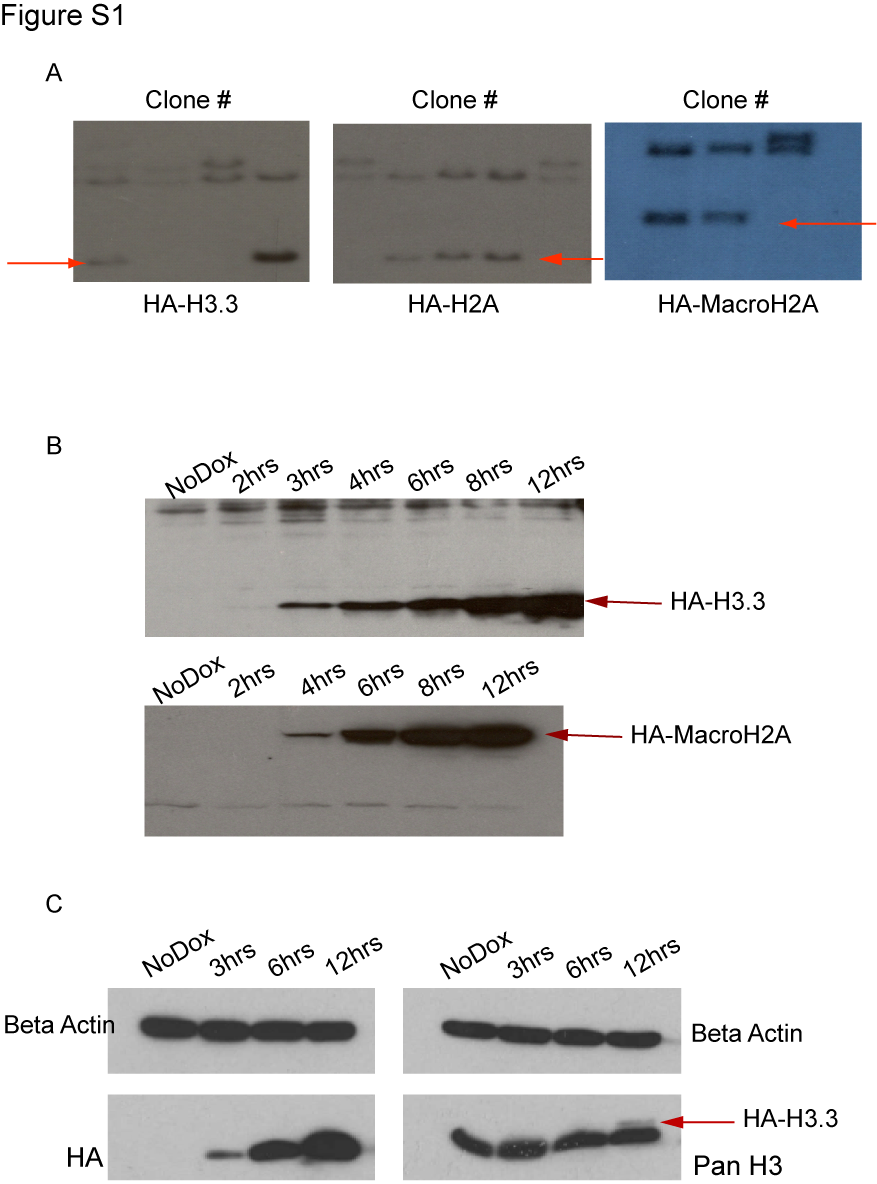

Supplement: Figure S1 — Validation of ES lines carrying inducible HA-tagged histones. (A) Southern blots showing correct integration of HA-histone constructs at the Col1A1 locus of KH2 cells. (B) Time course Western blots of HA-histone variant expression. As in Figure 1B . (C) Low levels of ectopic HA-H3.3 expression. Left and right sides show time courses of HA-H3.3 induction. Top panels show β-actin Western blots for loading controls, bottom panels show anti-HA (left) or anti-H3 (right) blots. Similar experiments were not feasible for Macro-H2A as the HA tag did not introduce a detectable mobility shift on western blot. (TIF) [file pgen.1004515.s001.tif]

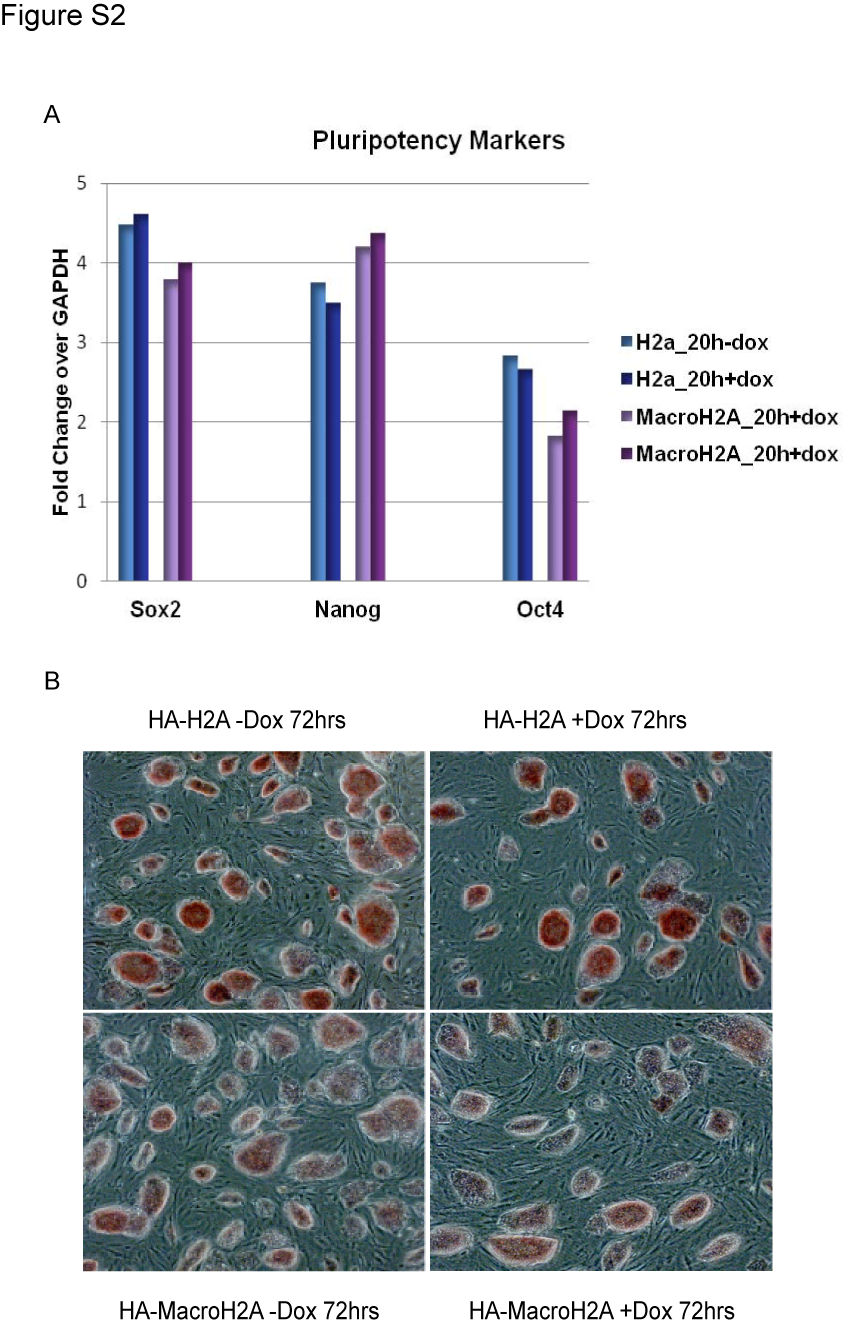

Supplement: Figure S2 — Ectopic HA-tagged histones do not affect ES pluripotency markers. (A) RNA levels of pluripotency markers. Q-RT-PCR for Oct4, Sox2, and Nanog before and after 20 hours of dox induction for the indicated histone variants. (B) Alkaline phosphatase staining, for uninduced and 72 hour induction for HA-H2A and HA-MacroH2A2. (TIF) [file pgen.1004515.s002.tif]

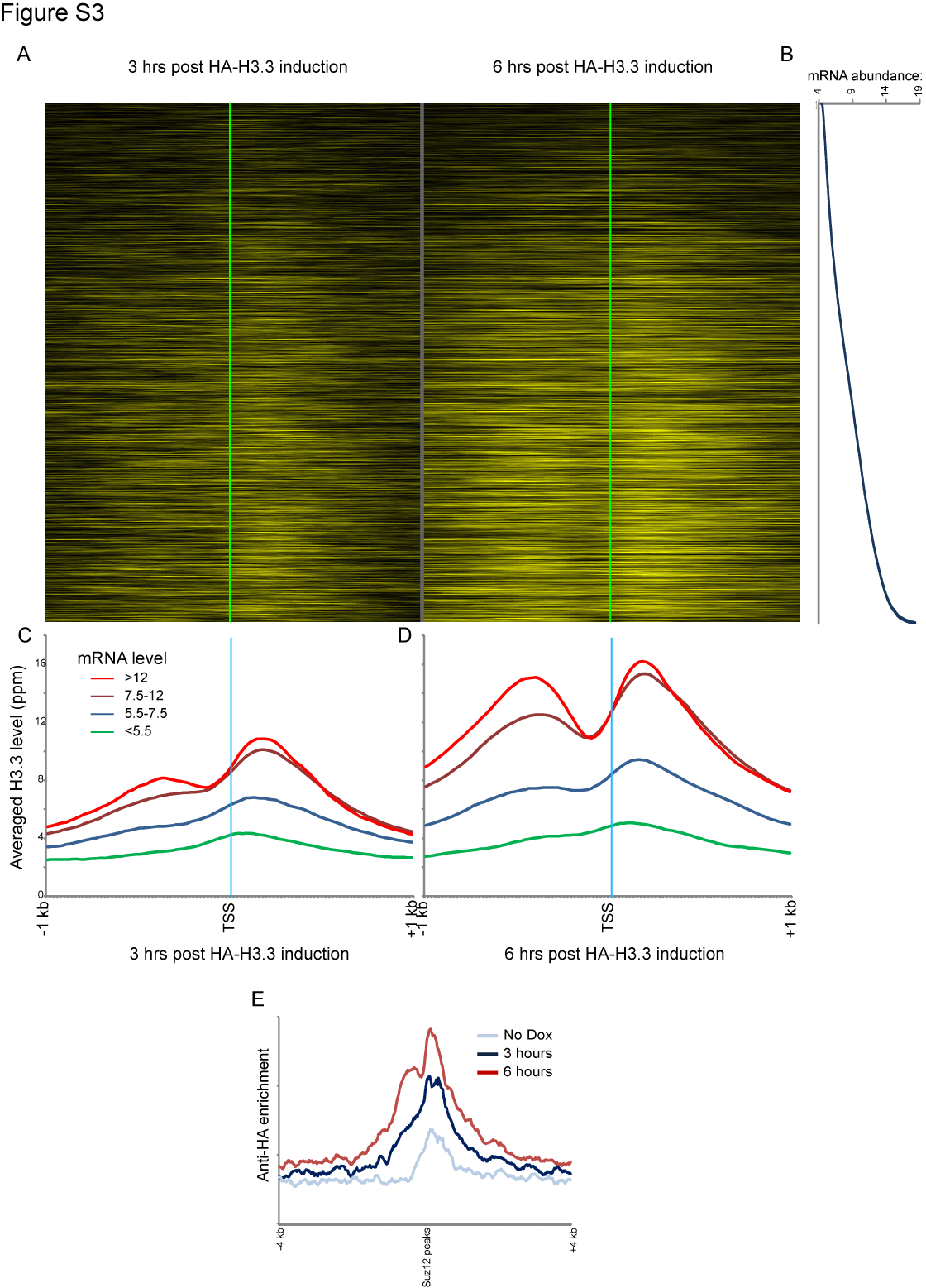

Supplement: Figure S3 — H3.3 dynamics. (A–B) HA-H3.3 mapping data for 3 and 6 hours after HA-H3.3 induction. ES cells carrying tet-inducible HA-H3.3 were subject to 3 or 6 hours of doxycycline, as indicated. TSS-aligned data are shown for all named genes (A), sorted according to expression level in ES cells (B). (C–D) Dynamic aspects of histone H3.3 replacement. Here, TSS-aligned ChIP-Seq data for HA-H3.3 are averaged for genes in each of four expression categories. Notably, highly-expressed genes show symmetric H3.3 peaks at 6 hours but show stronger downstream peaks at 3 hours, showing that steady-state mapping of H3.3 obscures subtleties of chromatin dynamics. In this regard our data subtly disagree with CATCH-IT metabolic labeling studies, which show more rapid overall protein dynamics upstream of the TSS than downstream [43]. This discrepancy could arise from the fact that CATCH-IT identifies replacement dynamics for ALL DNA-bound proteins, and this dataset explicitly focuses on H3.3, or may result from the fact that Yang et al do not analyze formaldehyde-crosslinked chromatin, whereas we use formaldehyde crosslinking. In any case, our observation of more rapid H3.3 replacement downstream of the TSS is consistent with the greater number of short transcripts generated downstream of promoters relative to upstream in mammals [44]. These results imply that under steady state mapping conditions (e.g. Goldberg et al), or after extended induction in a pulse-chase system (eg at 6 hours), nucleosomes exhibiting moderate to high turnover rates become saturated with H3.3. (E) Averaged anti-H3.3 data for the indicated Dox induction times, averaged for 8 kb surrounding Suz12 binding peaks [45]. (TIF) [file pgen.1004515.s003.tif]

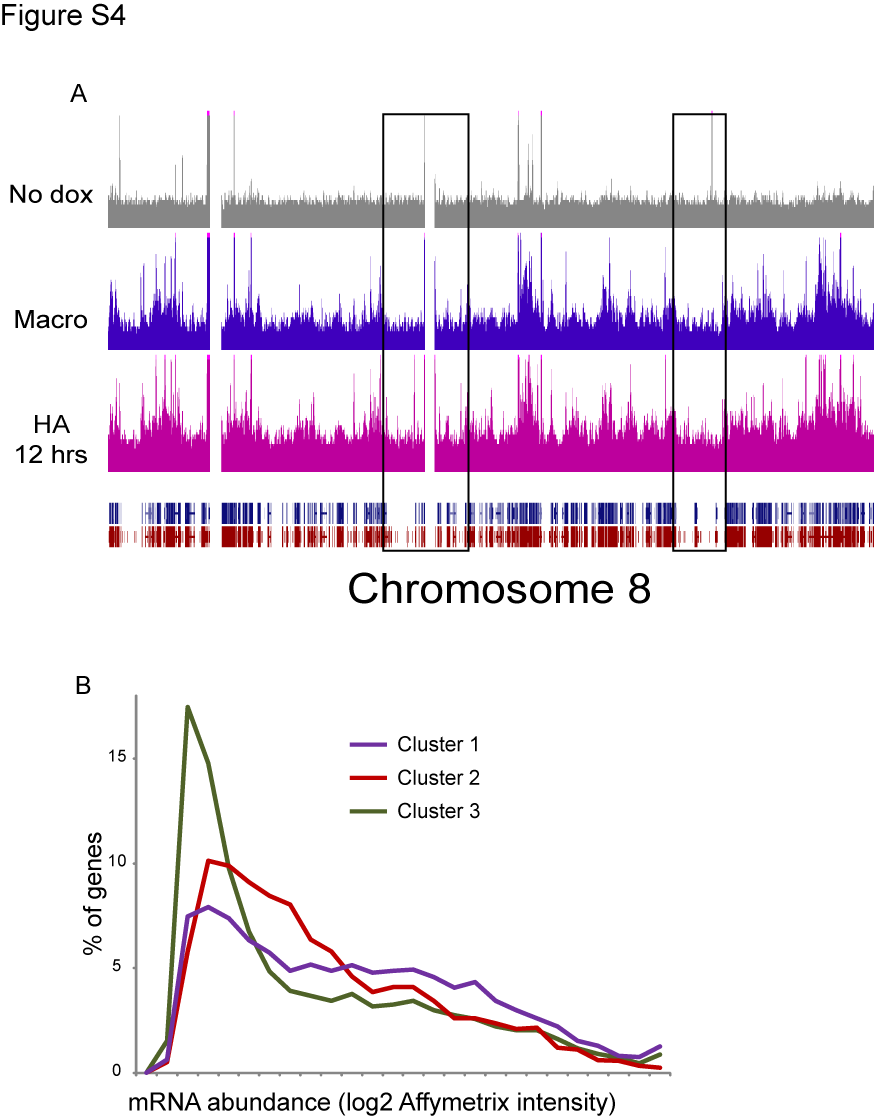

Supplement: Figure S4 — ES cell MacroH2A2 localizes to gene-rich regions. (A) As in Figure 2A , but for chromosome 8. (B) Histogram of mRNA abundances [42] for genes in each of the three clusters from Figure 2C . (TIF) [file pgen.1004515.s004.tif]

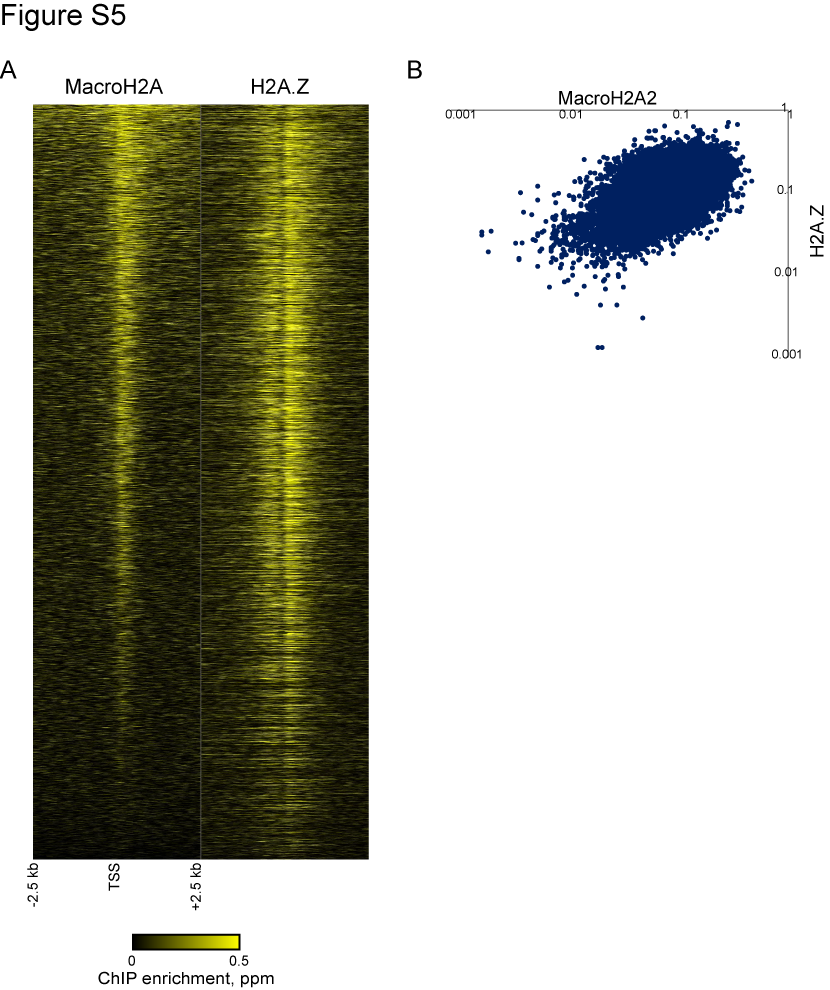

Supplement: Figure S5 — Comparison of MacroH2A2 and H2A.Z localization in ES cells. (A) Data for all named genes is shown for MacroH2A2 (this study) and H2A.Z [39], with genes sorted by MacroH2A2 level. (B) Scatterplot of promoter H2A variant enrichments. Enrichment for each variant was calculated as the average ChIP-Seq enrichment across 4 kB surrounding the TSS. (TIF) [file pgen.1004515.s005.tif]

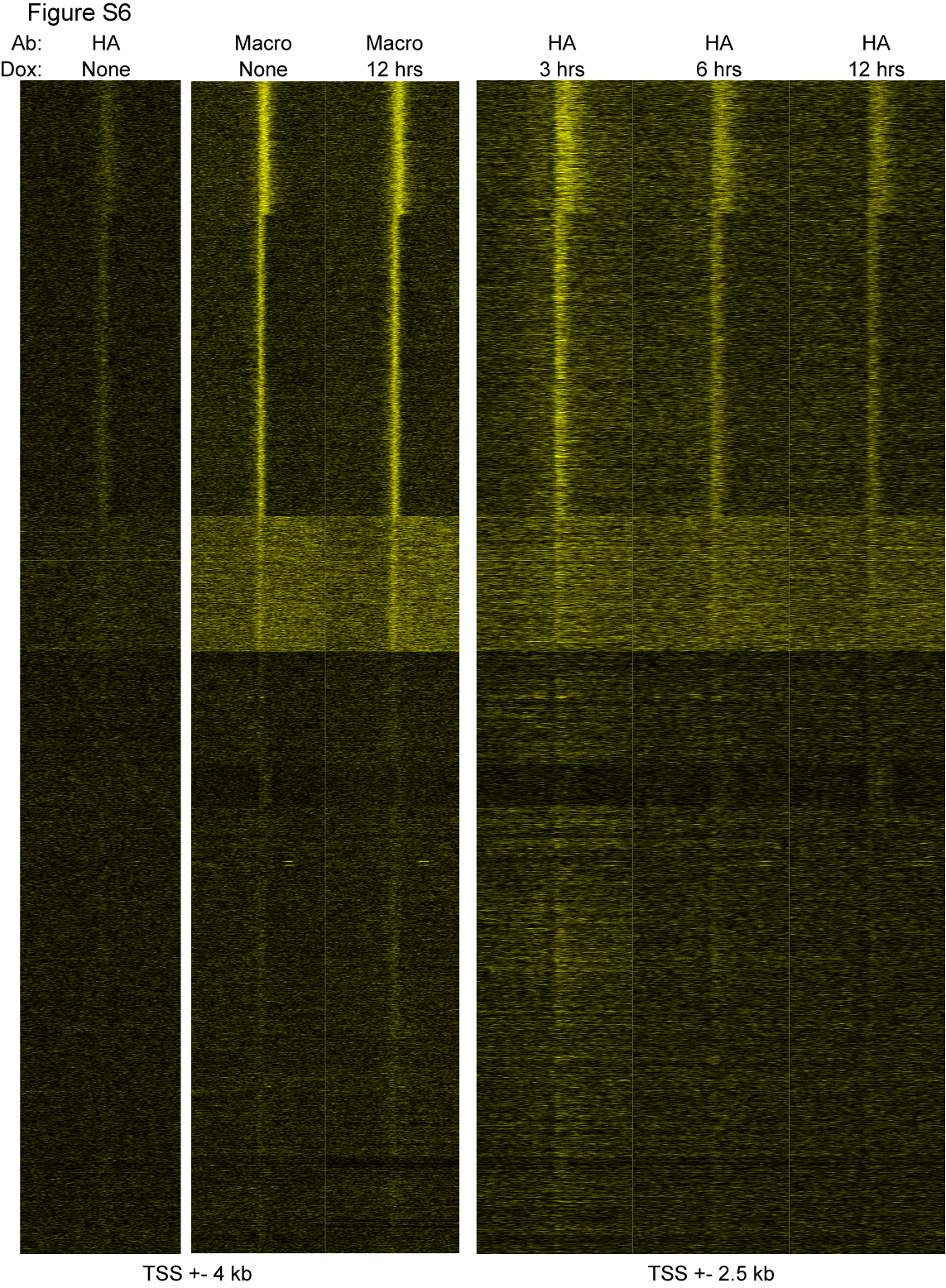

Supplement: Figure S6 — MAcroH2A2 localization in ES cells. Six panels show MacroH2A2 localization, or control, sorted according to K means clustering of anti-MacroH2A2 ChIP-Seq ( Figure 2C ) in ES cells. Panels show anti-HA or anti-MacroH2A2 datasets, as indicated, in tet-HA-MacroH2A2 cells induced with doxycycline for varying times as indicated. Note strong correlations between data from anti-Macro mapping and anti-HA mapping in induced cells. Signal is generally far lower in uninduced cells, although low level leaky expression presumably results in HA patterns similar to endogenous Macro localization. Alternatively, open chromatin may be more susceptible to artifactual isolation even in the absence of leaky HA expression. (TIF) [file pgen.1004515.s006.tif]

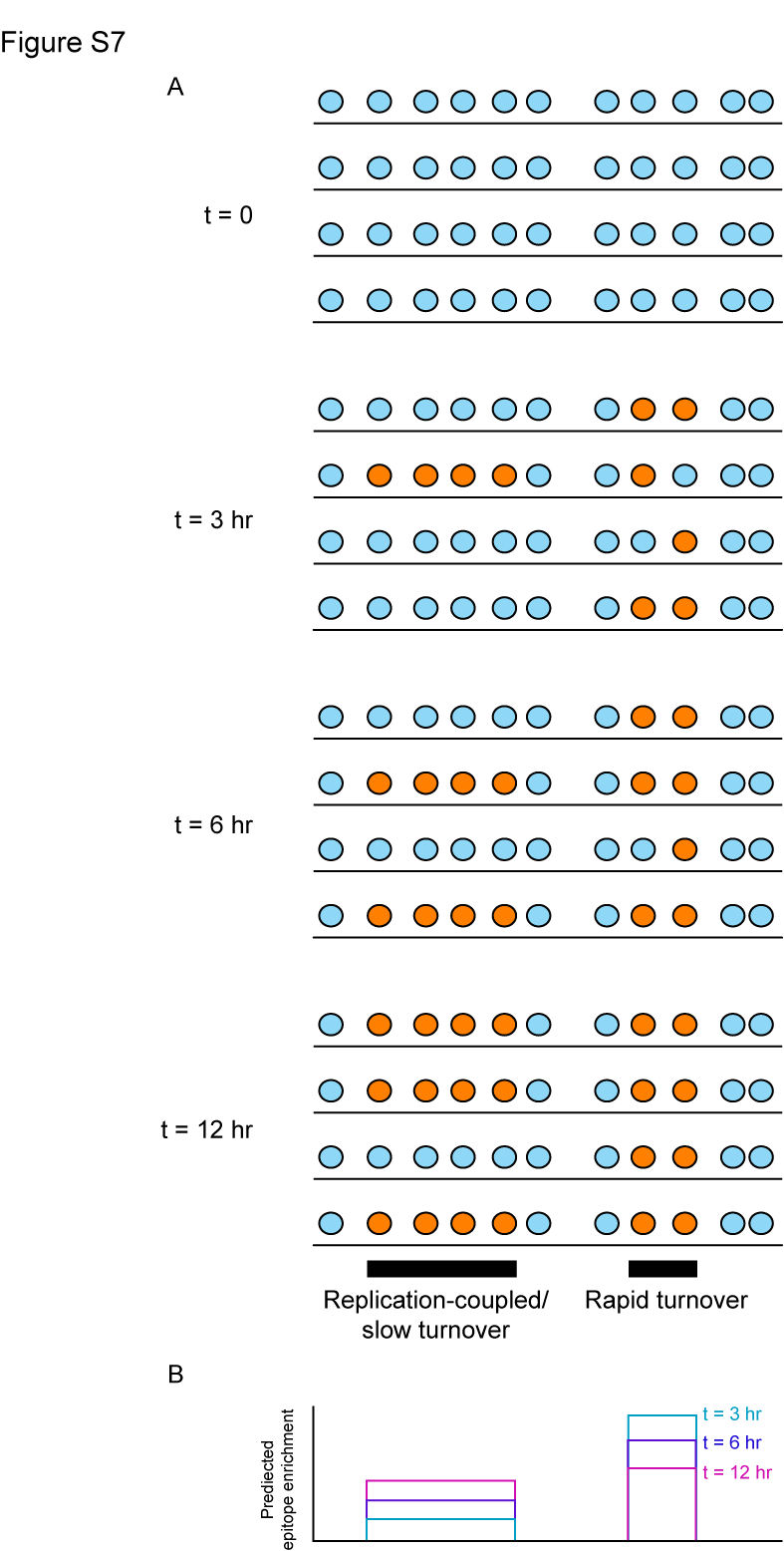

Supplement: Figure S7 — Expected time course behavior in asynchronous cells. (A) Cartoon of a genomic locus in a population of cells during a time course of epitope-tagged histone expression. Untagged nucleosomes are colored blue, epitope tagged-nucleosomes are colored orange. Each time point shows four loci, meant to correspond to four different cells in a population. Over time, the locus undergoing replication-coupled histone variant incorporation gains epitope tag gradually as cells asynchronously transit S phase. In contrast, the locus exhibiting rapid turnover gains epitope-tagged histones even at early time points. (B) Predicted behavior of ChIP-Seq at the locus shown in (A). Thanks to genome-wide normalization methods, the “hot” locus will exhibit very high relative epitope tag enrichment at earlier time points, but this peak will diminish in amplitude as slow turnover or replication-dependent incorporation occurs in an increasing fraction of cells, yielding a greater total number of loci carrying the epitope tag. Importantly, assessment of relatively hot and cold loci is totally insensitive to normalization method—an alternative normalization could be used in which hot loci are assumed to be saturated at early time points, and in this case the right peak would not change and the left peak would show more dramatic increases in enrichment over time. Yet calculating turnover by comparing data from t = 3 and t = 12 would nonetheless show the exact same difference when comparing the kinetic behavior of the right peak with the behavior of the left peak. (TIF) [file pgen.1004515.s007.tif]

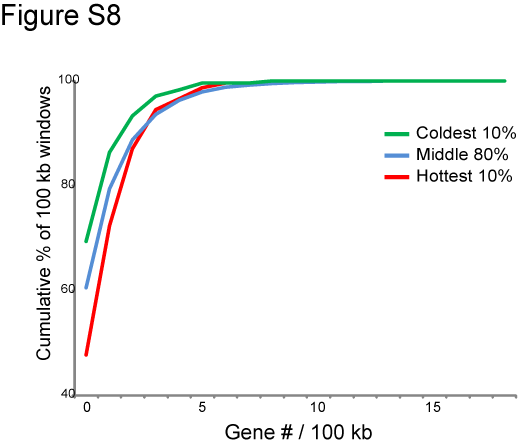

Supplement: Figure S8 — Rapid MacroH2A2 turnover in gene-rich regions. Cumulative distribution of gene richness (genes/100 kb tile, y axis), for 100 kb tiles grouped by hottest (top 10%), coldest (bottom 10%), and intermediate (remaining) Macro turnover behavior. (TIF) [file pgen.1004515.s008.tif]

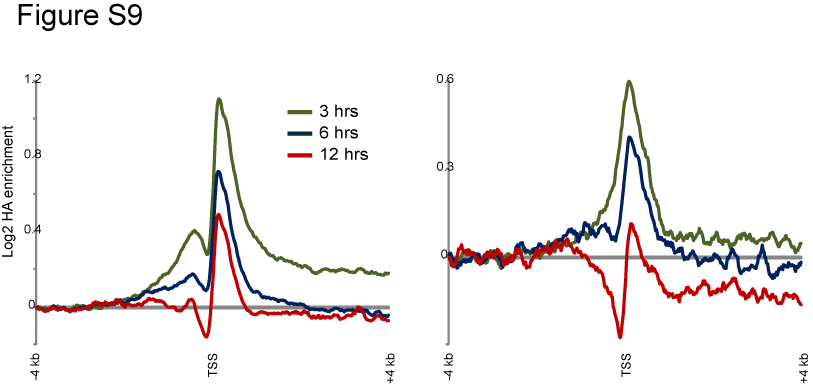

Supplement: Figure S9 — Reproducible MacroH2A2 dynamics. For both panels, genome-wide anti-HA datasets are averaged for all named genes at 3, 6 and 12 hours post-HA-MAcroH2A2 induction. Left panel shows more recent dataset used for analyses throughout manuscript, right panel shows prior dataset (which was undersequenced, and hence not used for additional analyses). In both cases, progressive “loss” of Macro over TSSs reveals rapid Macro dynamics at promoters in ES cells. (TIF) [file pgen.1004515.s009.tif]

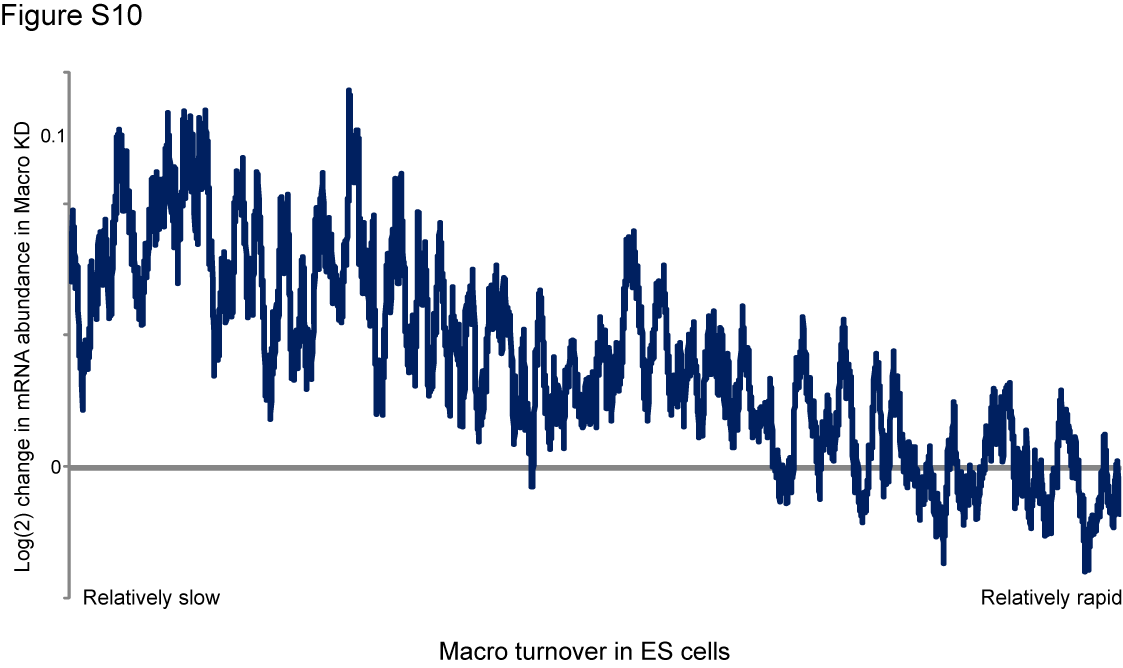

Supplement: Figure S10 — MacroH2A2 represses genes with stable MacroH2A2. mRNA abundance changes resulting from MacroH2A2 knockdown were measured by Affymetrix microarray (Table S4). Change in mRNA abundance is plotted on the y axis (positive values represent mRNA derepression upon knockdown), and genes are sorted on the x axis by inferred MacroH2A2 turnover, from slow to rapid replacement. mRNA abundance changes are shown as an 80 gene running window average. (TIF) [file pgen.1004515.s010.tif]

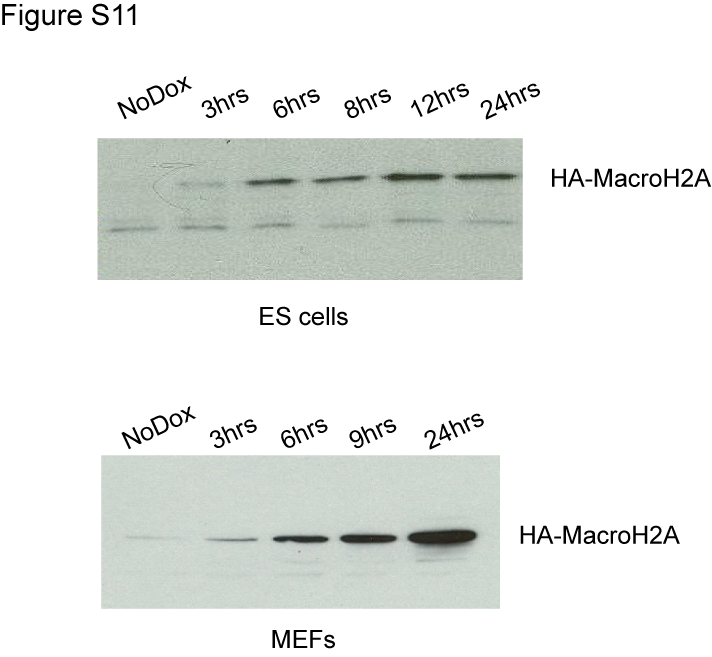

Supplement: Figure S11 — HA induction kinetics in ES and MEFs. Western blots showing anti-HA staining for the indicated time points of Dox induction in ES and MEF lines, as indicated. (TIF) [file pgen.1004515.s011.tif]

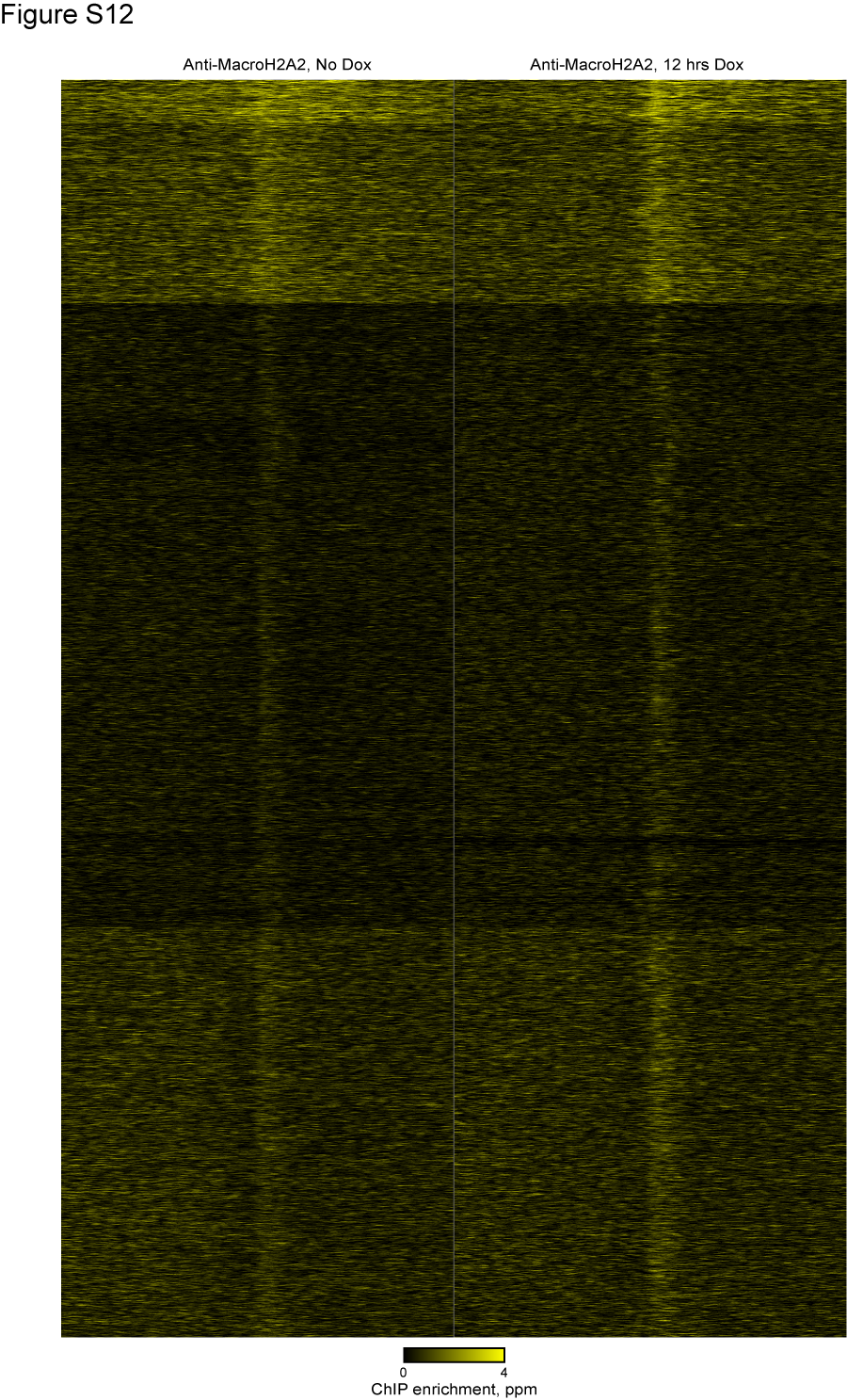

Supplement: Figure S12 — Reproducible MacroH2A2 localization in MEFs. Genome-wide anti-Macro localization patterns for all genes (k means clustered, k = 4) for tet-inducible HA-Macro MEFS without Dox (left panel), or after 12 hours of Dox (right panel). (TIF) [file pgen.1004515.s012.tif]

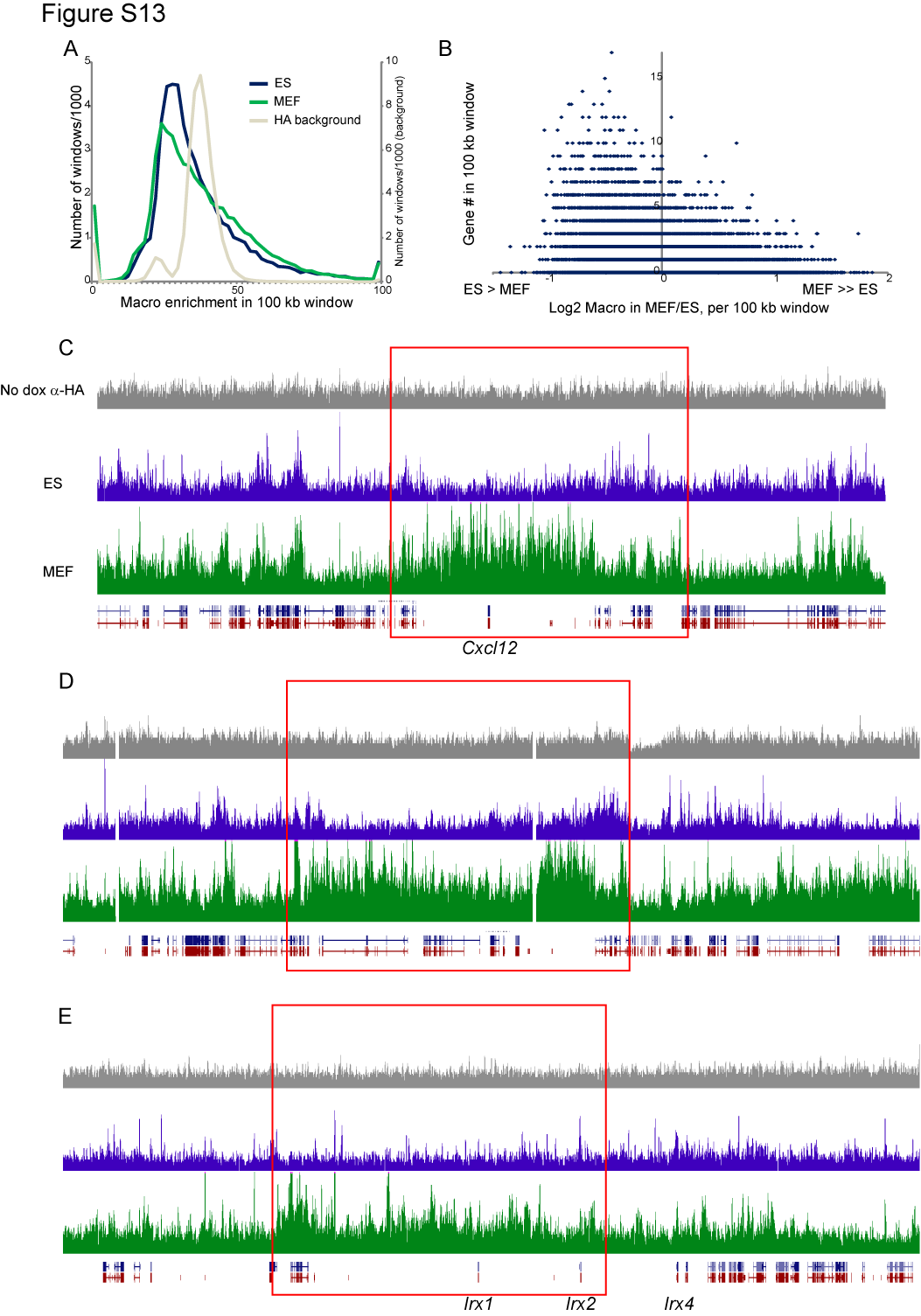

Supplement: Figure S13 — MacroH2A2 gain over gene-poor regions in MEFs. (A) Global changes in MacroH2A2 between ES cells and MEFs. Histogram of normalized MacroH2A2 enrichment (x axis) for 100 kb bins, shown for anti-MacroH2A2 ChIP-Seq from ES cells and MEFs, or for anti-HA ChIP-Seq from uninduced ES cells (grey). Note that anti-HA dataset is plotted on a different y axis scale (right). Increased right shift for MEFs is consistent with the known global gain in MacroH2A2 in this cell type relative to ES cells. (B) MacroH2A2 gain in MEFs primarily occurs in gene deserts. X axis shows changes in average MacroH2A2 enrichment between ES cells and MEFs – positive values represent gain in Macro in MEFs, negative values represent relative “loss” of Macro in MEFs. Y axis shows gene count per 100 kb window. Note that, due to increasing MacroH2A2 levels during differentiation (H2afy2 is upregulated 4-fold at the mRNA level between ES cells and MEFs), it is likely that the apparent “loss” of Macro over gene-rich regions reflects maintenance of Macro levels, whereas the gain in Macro over gene deserts shown here is being underestimated. (C–E) Examples of gene-poor regions with greater levels of Macro in MEFs than in ES cells. (TIF) [file pgen.1004515.s013.tif]

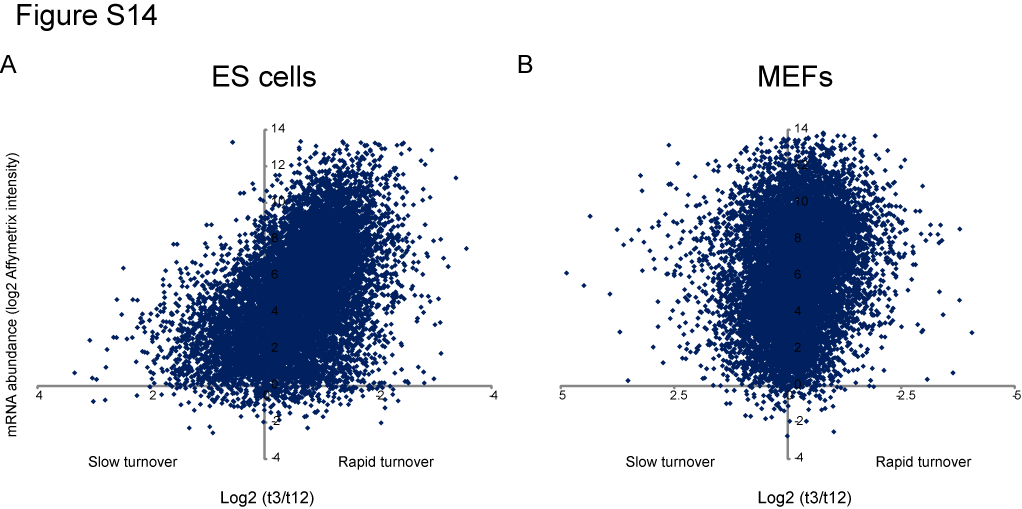

Supplement: Figure S14 — Macro dynamics are poorly-correlated with mRNA abundance in MEFs. (A–B) Scatterplot between inferred Macro dynamics (calculated as log2 of HA enrichment at 3 hours of Dox induction divided by HA levels at 12 hours), shown on the x axis, and mRNA abundance, on the y axis. Macro turnover in ES cells (A) shows a strong correlation between rapid turnover and high mRNA abundance, whereas this correlation is very weak in MEFs (B). (TIF) [file pgen.1004515.s014.tif]

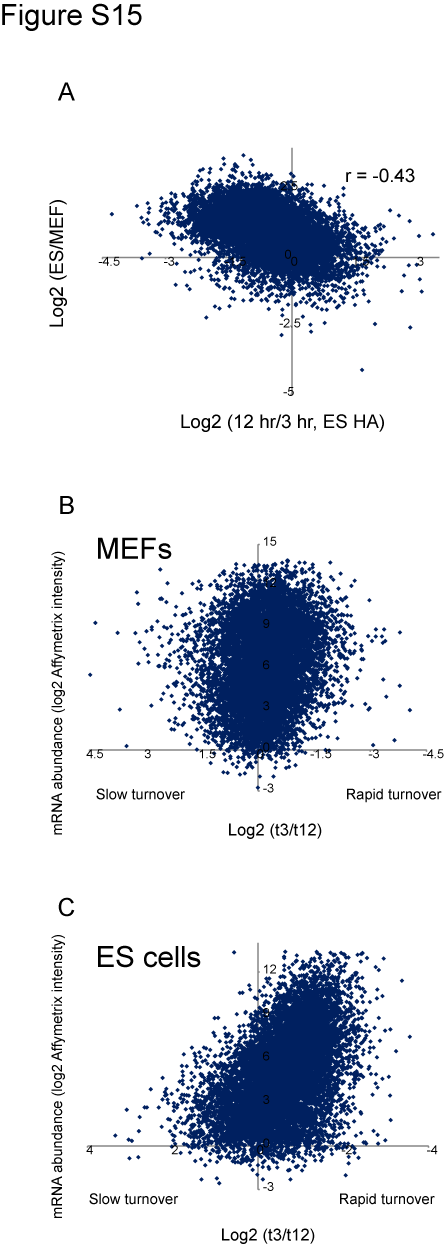

Supplement: Figure S15 — Macro differences between ES and MEFs do not result from X inactivation. (A–C) These panels reproduce Figures 6C , S14B, and S14A, respectively, but with all X-linked genes removed from the dataset. Other analyses are similarly unaffected by removal of X-linked genes. (TIF) [file pgen.1004515.s015.tif]

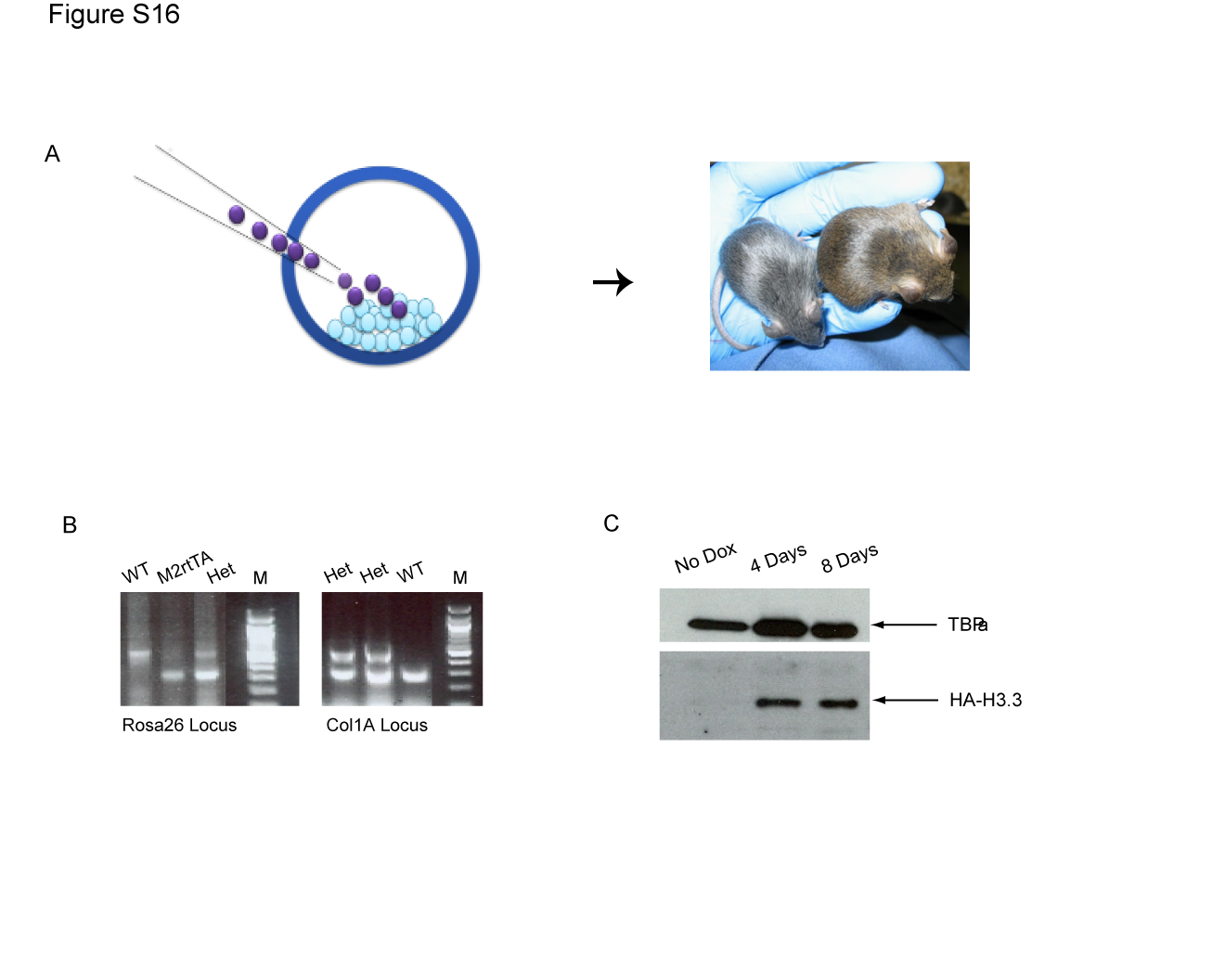

Supplement: Figure S16 — Generation of turnover mice. (A) Left side shows schematic of ES injection into blastocysts. Right panel shows offspring generated from Tet-HA-H3.3 injection with coat color indicating very high level of chimaerism. (B) Southern blots of offspring showing germline transmission of both loci required for Doxycycline induction of HA-tagged H3.3. (C) Western blots of nuclear extracts prepared from livers of animals provided with Dox for the indicated times, showing expected induction of HA-H3.3. (TIF) [file pgen.1004515.s016.tif]
